# Lipid Levels and Lung Cancer Risk: Findings from the Taiwan National Data Systems from 2012 to 2018

**DOI:** 10.1007/s44197-025-00351-8

**Published:** 2025-01-30

**Authors:** Jung-Yueh Chen, Nai-Hui Chi, Ho-Shen Lee, Chia-Ni Hsiung, Chang-Wei Wu, Kang-Chi Fan, Meng-Rui Lee, Jann-Yuan Wang, Chao-Chi Ho, Jin-Yuan Shih

**Affiliations:** 1 School of Medicine, College of Medicine, I-Shou University, Kaohsiung, Taiwan; 2 Department of Internal Medicine, E-DA Hospital, I-Shou University, Kaohsiung, Taiwan; 3 Department of Nursing, National Taiwan University Hospital, Taipei, Taiwan; 4 School of Medicine for International Students, College of Medicine, I-Shou University, Kaohsiung, Taiwan; 5 Program in Precision Medicine, National Tsing Hua University, Hsinchu, Taiwan; 6 Institute of Molecular Medicine, National Tsing Hua University, Hsinchu, Taiwan; 7 Department of Internal Medicine, National Taiwan University Hospital Hsinchu Branch, Hsinchu, Taiwan; 8 Department of Internal Medicine, National Taiwan University Hospital and College of Medicine, National Taiwan University, No.7, Chung Shan S. Rd., Zhongzheng District, Taipei City, 100225 Taiwan

**Keywords:** Lung cancer, Triglyceride, Total cholesterol, High-density lipoprotein, EGFR-mutated lung cancer

## Abstract

**Background:**

Lipids are known to be involved in carcinogenesis, but the associations between lipid profiles and different lung cancer histological classifications remain unknown.

**Methods:**

Individuals who participated in national adult health surveillance from 2012 to 2018 were included. For patients who developed lung cancer during follow-up, a 1:2 control group of nonlung cancer participants was selected after matching. Multivariate conditional logistic regression was used to explore the associations between lipid profiles, different lung cancer histological classifications and epidermal growth factor receptor mutation statuses. Subgroup, sensitivity, and dose‒response analyses were also performed.

**Results:**

A total of 4,704,853 participants (30,337 lung cancer participants and 4,674,516 nonlung cancer participants) were included. In both the main and sensitivity analyses, the associations remained constant between lower high-density lipoprotein (HDL) cholesterol levels and a higher risk of lung cancer (main analysis: odds ratio: 1.13 [1.08–1.18]) and squamous cell carcinoma (1.29 [1.16–1.43]). Hypertriglyceridemia was associated with a lower risk of adenocarcinoma (0.90 [0.84–0.96]) and a higher risk of small cell lung cancer (1.31 [1.11–1.55]). Hypercholesterolemia was associated with a lower risk of squamous cell carcinoma (0.84 [0.76–0.94]). In the subgroup analysis, lower HDL cholesterol levels were associated with greater risk across most subgroups. HDL cholesterol levels also demonstrated a dose‒response association with the development of lung cancer.

**Conclusions:**

The distinct associations between specific lipid profiles and lung cancer subtypes suggest that lipid metabolism may play different mechanistic roles in lung cancer development.

**Supplementary Information:**

The online version contains supplementary material available at 10.1007/s44197-025-00351-8.

## Introduction

Lipids are recognized primarily for their involvement in the development of atherosclerosis, and dyslipidemia represents a significant risk factor for cardiovascular and cerebrovascular diseases. However, lipids also play a role in carcinogenesis. Several mechanisms may account for this association. For example, lipids serve as a vital energy resource, and the rapid growth of cancer cells demands substantial amounts of energy and sterol metabolites [1]. Another potential mechanism linking lipids and cancer formation is the modulation of ferroptotic-mediated cell death, which is driven by iron-dependent lipid hydroperoxide formation [2]. Additionally, lipids contribute to the stabilization of tumor cell membranes and facilitate tumor metastasis [2, 3]. The mechanisms mentioned above may represent only a fraction of the intricate interactions between lipids and cancer.

While lipids are more closely associated with certain cancer types, such as breast cancer, colorectal cancer, and prostate cancer, the connection between lipids and lung cancer is not straightforward or well established. Nevertheless, previous reports have described the association between lung cancer and dyslipidemia. A meta-analysis that included 14,052 cases of lung cancer revealed a significant inverse association between total cholesterol levels and lung cancer risk, a negative association between high-density lipoprotein (HDL) cholesterol levels and lung cancer risk, and a positive association between total triglyceride levels and lung cancer risk [4]. Notably, previous studies have focused primarily on the overall risk of developing lung cancer in relation to lipid levels and have not explored the various subtypes of lungQ3 cancer. This is possibly due to the relatively small sample sizes in individual studies [5]. Therefore, further research on the complex relationship between lipids and lung cancer, particularly in the context of specific lung cancer cell types, is needed.

Elucidating the association between lipids and lung cancer can have a significant impact on population-based strategies for preventing lung cancer by altering the lipid profile. Therefore, we initiated a large-scale, population-based study to investigate the relationship between lipids and lung cancer. This study focused on different types of lung cancer and their epidermal growth factor receptor (EGFR) mutation status.

## Patients and Methods

### Study Design and Population

We extracted data from the National Adult Health Examination Database, the Taiwan Cancer Registry and the National Health Insurance database. The National Health Insurance Database includes health care-related data from all residents in Taiwan [6, 7]. The National Adult Health Examination Database contains data from nationwide free preventive health examinations for those aged 40 years or older; the goal of this program is to maintain the health of middle-aged and elderly individuals and to facilitate early detection, intervention, and treatment of chronic diseases [8]. The health check-ups provided included physical examinations, blood biochemistry tests, and health consultations (https://www.hpa.gov.tw/).

To confirm cancer diagnoses and collect information on histology classification and EGFR status, we utilized data from the Taiwan Cancer Registry. The Taiwan Cancer Registry is a government-maintained cancer registry that operates routinely, boasts high-quality data and encompasses more than 90% of all cancer patients in Taiwan (https://twcr.tw/) [6].

We recruited cancer-free participants who attended their first adult health examination between 2012 and 2018, with their baseline lipid profiles obtained during this examination. These participants were followed from their initial health examination until the end of 2018. For participants diagnosed with lung cancer during the follow-up period, we recruited a control group at a 1:2 ratio. This control group remained cancer-free at the time of diagnosis. The matching variables for the control group included age, sex, body mass index, and the year of their first health examination (Supplementary Fig. 1). In summary, the cases included participants who developed lung cancer after their baseline lipid profile examination, whereas the controls were participants who remained cancer-free throughout the follow-up period after their baseline lipid test.

The enrolled population was identified, and their basic profile, underlying diseases, and use of medication were determined from the National Health Insurance Database in Taiwan [6]. In addition, mortality information was obtained from the Department of Statistics, Taiwan [9].

### Data Collection and Definition

Additionally, we used the Charlson comorbidity index from the National Health Insurance Database to assess our patients’ comorbid conditions [10]. All patients with malignancy-associated scores were excluded, as in previous studies [11, 12]. Patients without comorbidities were defined as those who had no underlying disease (i.e., the Charlson comorbidity index score was zero). The histological classification and EGFR mutation status of lung cancer patients were determined on the basis of data from the Taiwan Cancer Registry.

Data on pharmaceutical agents were collected to examine their potential impact on the risk of lung cancer; if necessary, drug counts were adjusted. The drug counts that were adjusted in this study included the medications used to treat type 2 diabetes, hyperlipidemia, and hypertension as well as select antibiotics. We computed the defined daily dose for each Anatomical Therapeutic Chemicalcode and estimated the risk of lung cancer through univariate logistic regression. The cumulative effect of each drug was summarized, with weights assigned on the basis of beta values, defining our drug counts in relation to their impact on lung cancer.

The cutoff levels for an abnormal lipid profile were determined according to local guidelines and are as follows: Hypercholesterolemia is characterized by elevated total cholesterol and is defined as a total cholesterol level above 200 mg/dL. Hypertriglyceridemia is characterized by elevated triglycerides and is defined as a triglyceride level above 200 mg/dL. Hypoalphalipoproteinemia is characterized by a lower HDL cholesterol level and is defined as an HDL cholesterol level lower than 40 mg/dL. An elevated LDL cholesterol level was defined as an LDL cholesterol level above 160 mg/dL [13, 14]. Lipid data were obtained from the baseline lipid examination of participants when they first attended adult health examinations between 2012 and 2018.

### Statistical Analysis

Chi-square tests and Student’s t tests were used to compare categorical variables and continuous variables, respectively. After matching for age, sex, body mass index and year of examination, conditional logistic regression was used to explore the associations between lipid profiles and lung cancer. The following variables were adjusted in the conditional logistic regression: lipid profiles, smoking habits, alcohol consumption habits, drug counts, Charlson comorbidity index scores and comorbidities.

Subgroup analysis was performed on the basis of age, sex, body mass index, smoking habits, alcohol consumption habits, diabetes mellitus status and comorbidity status. Dose‒response analysis was performed by separating the lipid profile into quartiles and conducting restricted cubic spline regression. Spline regression models were employed to examine potential nonlinear relationships between lipid parameters and the incidence of lung cancer. The inclusion of spline terms allowed for the detection of inflection points or curvilinear trends in the data.

Sensitivity analysis was performed with Cox regression to test the robustness of our model and to exclude patients with lung cancer who were diagnosed within half a year after a health examination. Multivariate Cox regression was performed to explore the associations between lipids and lung cancer and was adjusted for confounders, including age, sex, body mass index, smoking habit, drinking habit, lipid profile, comorbidities and drug counts. All the statistical analyses were performed with SAS 9.4.

## Results

### Patient Characteristics

**Fig. 1:**
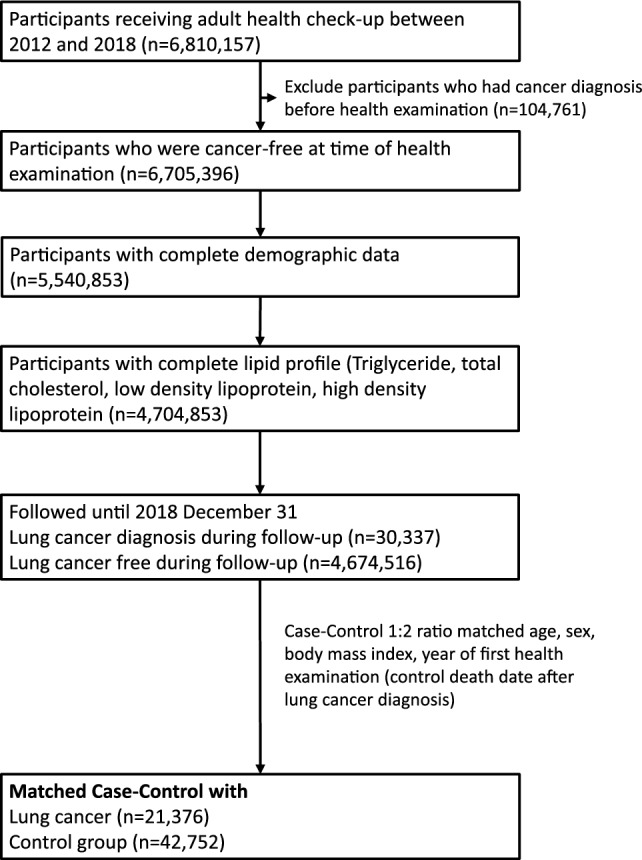
Flow chart of patients receiving adult health checkup recruitment from the Taiwan National Data Systems, 2012–2018

Figure 1 shows the flowchart of participant recruitment. A total of 4,704,853 participants, including 30,337 patients with lung cancer and 4,674,516 control participants, were recruited. The clinical characteristics of the participants are described in Supplementary Table 1. Among the 30,337 lung cancer patients, 20,107 (66.3%) had adenocarcinoma, 3958 (13.0%) had squamous cell carcinoma, 1989 (6.6%) had small cell lung cancer, and 4283 (14.1%) had other histological cell types. Across the histological classifications, the lung cancer patients in this study were mainly elderly patients, whereas the adenocarcinoma patients were predominantly younger and female.

Table 1 shows a 1:2 matched cohort of 21,376 lung cancer patients, including 13,282 (62.1%) patients with adenocarcinoma, 3389 (15.9%) patients with squamous cell carcinoma, 1713 (8.0%) patients with small cell lung cancer, 2992 (14.0%) patients with other types of lung cancer and 42,752 control participants. Before matching, the patients in the lung cancer group were older than those in the nonlung cancer group were (66.6 ± 11.2 vs. 57.8 ± 12.1 years, p < 0.0001), and there was a male preponderance in the former group (55.7% vs. 46.5%, p < 0.0001). Additionally, the lung cancer group had a slightly lower body mass index (24.4 ± 4.0 vs. 24.8 ± 4.0; p = 0.0015). Furthermore, patients in the lung cancer group were more likely to have lower HDL cholesterol levels (HDL cholesterol < 40 mg/dL, 19.5% vs. 16.6%, p < 0.0001). The nonlung cancer group was more likely to have hypercholesterolemia (45.5% vs. 42.4%, p < 0.0001) and hypertriglyceridemia (17.1% vs. 14.3%, p < 0.0001).

**Table 1:** Clinical characteristics of the unmatched and matched participants recruited from the Taiwan National Data Systems, 2012–2018

	Unmatched				Matched			
	All (N = 4,704,853)	Lung cancer (N = 30,337)	Control (N = 4,674,516)	p	All (N = 64,128)	Lung Cancer (N = 21,376)	Control (N = 42,752)	*p*
Age	57.9 ± 12.2	66.6 ± 11.2	57.8 ± 12.1	< 0.0001	65.6 ± 10.6	65.8 ± 10.4	65.7 ± 10.9	0.4358
30–50 yrs	1,351,009 (28.7)	2085 (6.9)	1,348,924 (28.9)	< 0.0001	2547 (4.0)	849 (4.0)	1698 (4.0)	1
50–60 yrs	1,409,598 (30.0)	6287 (20.7)	1,403,311 (30.0)		15,885 (24.8)	5295 (24.8)	10,590 (24.8)	
60–70 yrs	1,119,896 (23.8)	9548 (31.5)	1,110,348 (23.8)		24,666 (38.5)	8222 (38.5)	16,444 (38.5)	
> 70 yrs	824,350 (17.5)	12,417 (40.9)	811,933 (17.4)		21,030 (32.8)	7010 (32.8)	14,020 (32.8)	
Male	2,189,817 (46.5)	16,906 (55.7)	2,172,911 (46.5)	< 0.0001	45,000 (70.2)	15,000 (70.2)	30,000 (70.2)	1
Body Mass Index	24.8 ± 4.0	24.4 ± 4.0	24.8 ± 4.0	0.0015	24.4 ± 3.8	24.4 ± 3.7	24.4 ± 4.1	0.3693
< 18	99,903 (2.1)	772 (2.5)	99,131 (2.1)	< 0.0001	1408 (2.2)	523 (2.5)	885 (2.1)	0.6012
18–24	2,034,600 (43.2)	14,203 (46.8)	2,020,397 (43.2)		29,592 (46.2)	9922 (46.4)	19,670 (46.0)	
> 24	2,570,350 (54.6)	15,362 (50.6)	2,554,988 (54.7)		33,128 (51.7)	10,931 (51.1)	22,197 (51.9)	
Smoking								
No	4,022,121(85.7)	24,311 (80.1)	3,997,810 (85.5)	< 0.0001	52,491 (81.9)	16,212 (75.8)	36,279 (84.9)	< 0.0001
Seldom	138,262(2.9)	1115 (3.7)	137,147 (2.9)		2389 (3.8)	952 (4.5)	1437 (3.4)	
< one pack per day	368,907 (7.8)	3176 (10.5)	365,731 (7.8)		6151 (9.6)	2733 (12.8)	3418 (8.0)	
> one pack per day	175,563 (3.7)	1735 (5.7)	173,828 (3.7)		3097 (4.8)	1479 (6.9)	1618 (3.8)	
Drinking								
No	3,895,432 (82.8)	25,085 (82.7)	3,870,347 (82.8)	< 0.0001	52,048 (81.2)	17,056 (79.8)	34,992 (81.9)	< 0.0001
Occasionally	663,761 (14.1)	4068 (13.4)	659,693 (14.1)		9673 (15.1)	3336 (15.6)	6337 (14.8)	
Frequently	145,660 (3.1)	1184 (3.9)	144,476 (3.1)		2407 (3.8)	984 (4.6)	1423 (3.3)	
Hypertension								
SBP < 140 and DBP < 90 mmHg	3,168,723 (67.4)	19,087 (62.9)	3,149,636 (67.4)	< 0.0001	40,758 (63.6)	13,486 (63.1)	27,272 (63.8)	0.0758
SBP ≥ 140 or DBP ≥ 90 mmHg	1,536,130 (32.7)	11,250 (37.1)	1,524,880 (32.6)		23,370 (36.4)	7890 (36.9)	15,480 (36.2)	
Total cholesterol	197.8 ± 40.5	194.7 ± 40.5	197.8 ± 40.5	< 0.0001	194.7 ± 40.5	195.6 ± 40.8	194.3 ± 40.3	< .0001
Total cholesterol < 200 mg/dl	2,565,301 (54.5)	17,488 (57.65)	2,547,813 (54.5)	< 0.0001	36,645 (57.1)	12,524 (58.6)	24,121 (56.4)	< 0.0001
Total cholesterol ≥ 200 mg/dl	2,139,552 (45.5)	12,849 (42.35)	2,126,703 (45.5)		27,483 (42.9)	8852 (41.4)	18,631 (43.6)	
Triglycerides	144.5 ± 138.2	136.4 ± 126.3	144.6 ± 138.3	< 0.0001	138.2 ± 127.3	138.9 ± 133.3	137.9 ± 124.2	0.3748
Triglycerides < 200 mg/dl	3,900,173 (82.9)	25,989 (85.7)	3,874,184 (82.9)	< 0.0001	54,314 (84.7)	18,119 (84.8)	36,195 (84.7)	0.7388
Triglycerides ≥ 200 mg/dl	804,680 (17.1)	4348 (14.3)	800,332 (17.1)		9814 (15.3)	3257 (15.2)	6557 (15.3)	
HDL	54.1 ± 17.4	52.8 ± 17.2	54.1 ± 17.4	< 0.0001	52.2 ± 17.0	51.8 ± 17.2	52.4 ± 16.9	< 0.0001
HDL ≥ 40 mg/dl	3,923,169 (83.4)	24,422 (80.5)	3,898,747 (83.4)	< 0.0001	51,239 (79.9)	16,738 (78.3)	34,501 (80.7)	< 0.0001
HDL < 40 mg/dl	781,684 (16.6)	5915 (19.5)	775,769 (16.6)		12,889 (20.1)	4638 (21.7)	8251 (19.3)	
LDL	114.0 ± 40.6	113.3 ± 39.2	114.0 ± 40.6	< 0.0001	113.2 ± 39.2	112.8 ± 39.7	113.7 ± 38.9	*0.056*
LDL < 160 mg/dl	4,219,581 (89.7)	27,268 (89.9)	4,192,313 (89.7)	0.2555	57,663 (89.9)	19,253 (90.1)	38,410 (89.8)	*0.3733*
LDL ≥ 160 mg/dl	485,272 (10.3)	3069 (10.1)	482,203 (10.3)		6465 (10.1)	2123 (9.9)	4342 (10.2)	
Charlson Comorbidity Index	0.33 ± 0.82	0.42 ± 0.96	0.33 ± 0.82	< 0.0001	0.38 ± 0.89	0.43 ± 0.98	0.35 ± 0.85	< .0001

*CI* confidence interval; *DBP* diastolic blood pressure; *HDL* high-density lipoprotein; *LDL* low-density lipoprotein; *SBP* systolic blood pressure

After matching for age, sex and body mass index, the lung cancer group was still more likely to have a smoking habit (never smoked or active smoker, 24.2% vs. 15.1%, p < 0.0001), a drinking habit (occasional or frequent drinker, 20.2% vs. 18.1%, p < 0.0001) and a higher Charlson comorbidity index score (0.43 ± 0.98 vs. 0.35 ± 0.85, p < 0.0001). Participants with lung cancer were more likely to have lower HDL cholesterol levels (HDL cholesterol < 40 mg/dl, 21.7% vs. 19.3%, p < 0.0001), and those without lung cancer were more likely to have hypercholesterolemia (total cholesterol ≥ 200 mg/dl, 43.6% vs. 41.4%, p < 0.0001). Triglyceride levels (triglycerides ≥ 200 mg/dl, 15.2% vs. 15.3%, p = 0.7388) and LDL cholesterol levels (LDL cholesterol ≥ 160 mg/dl, 9.9% vs. 10.2%, p = 0.3733) were not different between the two groups. Other information, including the year of initial health examination, comorbidities and drug counts, is recorded in Supplementary Table 2.

The characteristics of the patients in the lung adenocarcinoma, squamous cell carcinoma, small cell lung cancer, and EGFR-mutated lung cancer groups and their control groups are described in Table 2. After matching, age, sex, year of health examination, and body mass index were not different between the control group and the lung cancer group with different histological classifications. Other information, including the year of initial health examination, comorbidities and drug counts, is recorded in Supplementary Table 3.

**Table 2:** Clinical characteristics of the matched lung cancer patients and controls across different histological classifications recruited from the Taiwan National Data Systems, 2012–2018

	All (N = 39,846)	Adenocarcinoma (N = 13,282)	Control (N = 26,564)	All (N = 10,167)	Squamous (N = 3389)	Control (N = 6778)	All (N = 13,986)	EGFR mutation (N = 4662)	Control (N = 9324)	All (N = 5139)	Small cell (N = 1713)	Control (N = 3426)
Age	63.9 ± 10.2	64.0 ± 9.9	63.9 ± 10.3	69.7 ± 10.7	69.8 ± 10.0	69.6 ± 11.0	63.6 ± 9.8	63.9 ± 9.4	63.7 ± 10.0	67.9 ± 10.9	68.0 ± 10.3	67.8 ± 11.2
30–50 yrs	4.3	4.3	4.3	2.8	2.8	2.8	3.5	3.5	3.52	3.7	3.7	3.7
50–60 yrs	29.5	29.5	29.5	13.4	13.4	13.4	30.7	30.7	30.7	17.7	17.7	17.7
60–70 yrs	41.8	41.8	41.8	31.37	31.4	31.4	43.5	43.5	43.5	34.7	34.7	34.7
> 70 yrs	24.4	24.4	24.4	52.46	52.5	52.5	22.3	22.3	22.3	43.8	43.8	43.8
Male	59.9	59.9	59.9	94.1	94.1	94.1	52.9	52.9	52.9	94.7	94.7	94.7
Body mass index	24.4 ± 3.7	24.4 ± 3.6	24.4 ± 3.7	24.3 ± 3.8	24.3 ± 3.9	24.3 ± 2.7	24.5 ± 3.6	24.4 ± 3.5	24.5 ± 3.7	24.7 ± 5.0	24.8 ± 7.0	24.6 ± 3.7
< 18	2.2	2.1	2.2	2.7	3.0	2.6	1.9	1.7	2.1	2.3	2.4	2.3
18–24	46.7	46.7	46.7	46.9	46.7	47.0	46.2	46.4	46.0	43.6	43.4	43.7
> 24	51.1	51.2	51.1	50.4	50.4	50.4	51.9	51.9	51.9	54.1	54.2	54.1
Smoking												
No	84.7	82.3***	85.9	75.6	62.5***	82.2	87.1	87.1	87.1	72.6	54.1***	81.8
Seldom	3.2	3.4	3.1	5.0	6.8	4.1	2.8	3.0	2.8	5.4	7.7	4.2
< one pack per day	8.1	9.4	7.5	12.8	19.7	9.4	6.8	6.4	6.9	14.2	24.5	9.1
> one pack per day	4.0	4.9	3.6	6.6	11.1	4.3	3.4	3.6	3.2	7.8	13.7	4.9
Drinking												
No	82.5	81.3***	83.1	77.8	75.7***	78.9	83.4	83.0	84.0	77.2	74.4***	78.6
Occasionally	14.2	14.9	13.8	17.5	17.9	17.3	13.3	13.7	13.1	17.9	18.6	17.5
Frequently	3.4	3.8	3.1	4.7	6.4	3.8	3.0	3.3	2.9	4.9	6.9	3.9
Hypertension												
SBP < 140 and DBP < 90 mmHg	65.0	64.3*	65.4	59.7	59.8	59.7	64.7	63.0**	65.5	61.4	59.5	62.3
SBP ≥ 140 or DBP ≥ 90 mmHg	35.0	35.7	34.6	40.3	40.3	40.3	35.1	37.0	34.5	38.7	40.5	37.7
Total cholesterol	197.2 ± 40.5	196.6 ± 40.4*	197.5 ± 40.5	188.5 ± 39.3	185.7 ± 39.5***	189.9 ± 39.1	198.6 ± 40.7	198.3 ± 40.5	198.8 ± 40.7	190.4 ± 41.3	190.5 ± 39.6	190.1 ± 44.4
Total cholesterol < 200 mg/dl	54.7	55.4	54.4	63.8	66.7***	62.4	53.5	54.0	53.2	62.5	64.1	61.7
Total cholesterol ≥ 200 mg/dl	45.3	44.6	45.6	36.2	33.3	37.6	46.6	46.0	46.8	37.5	35.9	38.3
Triglycerides	137.6 ± 121.1	135.8 ± 107*	138.4 ± 127.3	137.1 ± 129.2	139.2 ± 134.6	136.0 ± 126.4	137.6 ± 128.3	134.1 ± 103.4**	139.3 ± 139.0	149.0 ± 144.3	161 ± 248**	143 ± 127.7
Triglycerides < 200 mg/dl	84.9	85.5**	84.6	84.7	84.2	85.0	84.9	86.0*	84.4	82.2	78.9***	83.8
Triglycerides ≥ 200 mg/dl	15.1	14.5	15.5	15.3	15.8	15.0	15.1	14.0	15.6	17.8	21.1	16.2
HDL	53.2 ± 17.2	53.0 ± 17.1	53.3 ± 17.2	49.9 ± 16.2	48.9 ± 16.5***	50.4 ± 16.0	53.9 ± 17.0	54.8 ± 16.8	54.0 ± 17.2	49.8 ± 17.3	49.1 ± 19.2	50.1 ± 16.2
HDL ≥ 40 mg/dl	81.8	81.3	82.0	75.3	71.4***	77.3	83.2	83.1	83.3	74.1	71.5**	75.4
HDL < 40 mg/dl	18.2	18.7	18.0	24.7	28.6	22.7	16.8	16.9	16.8	25.9	28.5	24.6
LDL	115.0 ± 39.4	115.1 ± 40.0	115 ± 39.0	109.6 ± 37.1	107.4 ± 38.1***	110.7 ± 37.6	115.7 ± 40.4	116.5 ± 42.8	115.3 ± 39.1	109.9 ± 40.8	108.4 ± 40.8	110.6 ± 40.8
LDL < 160 mg/dl	89.2	89.4	89.1	91.8	92.2	91.6	88.93	88.57	89.11	91.2	90.6	91.4
LDL ≥ 160 mg/dl	10.8	10.7	10.9	8.2	7.8	8.4	11.07	11.43	10.89	8.9	9.4	8.6
Charlson Comorbidity Index	0.4 ± 0.9	0.4 ± 1.0***	0.3 ± 0.8	0.4 ± 1.0	0.5 ± 1.1***	0.4 ± 0.9	0.3 ± 0.8	0.4 ± 0.9	0.3 ± 0.8	0.4 ± 0.9	0.5 ± 1.0**	0.4 ± 0.9

*CI* confidence interval; *COPD* chronic obstructive pulmonary disease; *DBP* diastolic blood pressure; *HDL* high-density lipoprotein; *LDL* low-density lipoprotein; *SBP* systolic blood pressure

The data are presented as means ± standard deviations for continuous variables and percentages for categorical variables

*p < 0.05

**p < 0.01

***p < 0.001

### Associations Between Lipid Levels and Lung Cancer Risk

Table 3 shows the associations between different lipid levels and the risk of lung cancer, including the EGFR mutation status and different histological cell types.

**Table 3:** Associations between lipid profiles and lung cancer risk according to conditional logistic regression of the Taiwan National Data Systems, 2012–2018

	Lung cancer (All)			Adenocarcinoma			Squamous carcinoma			Small cell			EGFR mutation		
	OR	95% CI	p value	OR	95% CI	p value	OR	95% CI	p value	OR	95% CI	p value	OR	95% CI	p value
Triglyceride (mg/dl)	0.93	0.89–0.98	0.0055*	0.90	0.84–0.96	0.0006**	0.95	0.84–1.08	0.4318	1.31	1.11–1.55	0.0018**	0.87	0.78–0.96	0.0082*
Total Cholesterol (mg/dl)	0.94	0.90–0.97	0.0006*	0.98	0.93–1.02	0.2711	0.84	0.76–0.94	0.0011**	0.83	0.72–0.96	0.0133*	0.96	0.89–1.04	0.3388
HDL (mg/dl)	1.13	1.08–1.18	< 0.0001***	1.07	1.01–1.13	0.0026*	1.29	1.16–1.43	< 0.001***	1.08	0.93–1.25	0.3241	1.05	0.95–1.16	0.3473
LDL (mg/dl)	1.02	0.96–1.09	0.4526	1.00	0.93–1.07	0.9127	1.06	0.89–1.25	0.5364	1.21	0.96–1.3	0.1154	1.10	0.97–1.26	0.1545

*CI* confidence interval; *EGFR* epidermal growth factor receptor; *HDL* high-density lipoprotein; *LDL* low-density lipoprotein; *OR* odds ratio

*Significant in primary analysis

**Significant in primary analysis and sensitivity analysis

***Significant in primary analysis, sensitivity analysis and cubic spline regression analysis

A lower HDL level was associated with greater risks of lung cancer (1.13 [1.08–1.18], p < 0.0001), lung adenocarcinoma (1.07 [1.01–1.13], p = 0.0011), and lung squamous cell carcinoma (1.29 [1.16–1.43], p < 0.0001).

Hypercholesterolemia was associated with lower risks of squamous cell carcinoma (0.84 [0.76–0.94], p = 0.0011) and small cell lung cancer (0.83 [0.72–0.96], p = 0.0133).

Hypertriglyceridemia was associated with lower risks of lung cancer (0.93 [0.89–0.98], p = 0.0005), lung adenocarcinoma (0.90 [0.84–0.96], p = 0.0006) and EGFR-mutated lung cancer (0.97 [0.78–0.96], p = 0.0082) as well as a greater risk of small-cell lung cancer (1.31 [1.11–1.55], p = 0.0018).

### Sensitivity Analysis After Patients with Lung Cancer Diagnosed Within Half a Year After an Adult Health Examination were Excluded

After patients who were diagnosed with lung cancer within half a year after an adult health examination were excluded, a lower HDL cholesterol level was still associated with a greater risk of lung cancer (1.06 [1.01–1.10], p = 0.0401) and squamous cell carcinoma (1.12 [1.00–1.26], p = 0.0491).

Hypercholesteremia was associated with a lower risk of squamous cell carcinoma (0.88 [0.79–0.99], p = 0.0283).

Hypertriglyceridemia was associated with a lower risk of adenocarcinoma (0.90 [0.84–0.97], p = 0.0049) and a higher risk of small cell lung cancer (1.40 [1.16–1.69], p = 0.0005) (Supplementary Table 4).

### Cox Regression Model

The results of the Cox regression model revealed that lower HDL cholesterol levels were associated with a greater risk of overall lung cancer (1.11 [1.08–1.15]), adenocarcinoma (1.08 [1.04–1.12]), squamous cell carcinoma (1.21 [1.12–1.31]) and EGFR-mutated lung cancer (1.13 [1.08–1.20]). Hypercholesteremia was associated with a lower risk of overall lung cancer (0.95 [0.93–0.98]), squamous cell carcinoma (0.87 [0.83–0.92]) and small cell lung cancer (0.83 [0.74–0.92]). Hypertriglyceridemia was associated with lower risks of overall lung cancer (HR: 0.88 95% CI: 0.83–0.93), adenocarcinoma (0.86 [0.82–0.90]), and EGFR-mutated lung cancer (0.87 [0.83–0.92]) and a greater risk of small cell lung cancer (1.25 [1.11–1.41]). Higher LDL was associated with higher risks of developing small cell lung cancer (1.28 [1.08–1.52]) and EGFR-mutated (1.08 [1.02–1.16]) lung cancer. The results of the Cox regression model analysis are presented in Supplementary Table 5.

### Subgroup Analysis on the Basis of Different Lipid Levels and Lung Cancer Subtypes

**Fig. 2:**
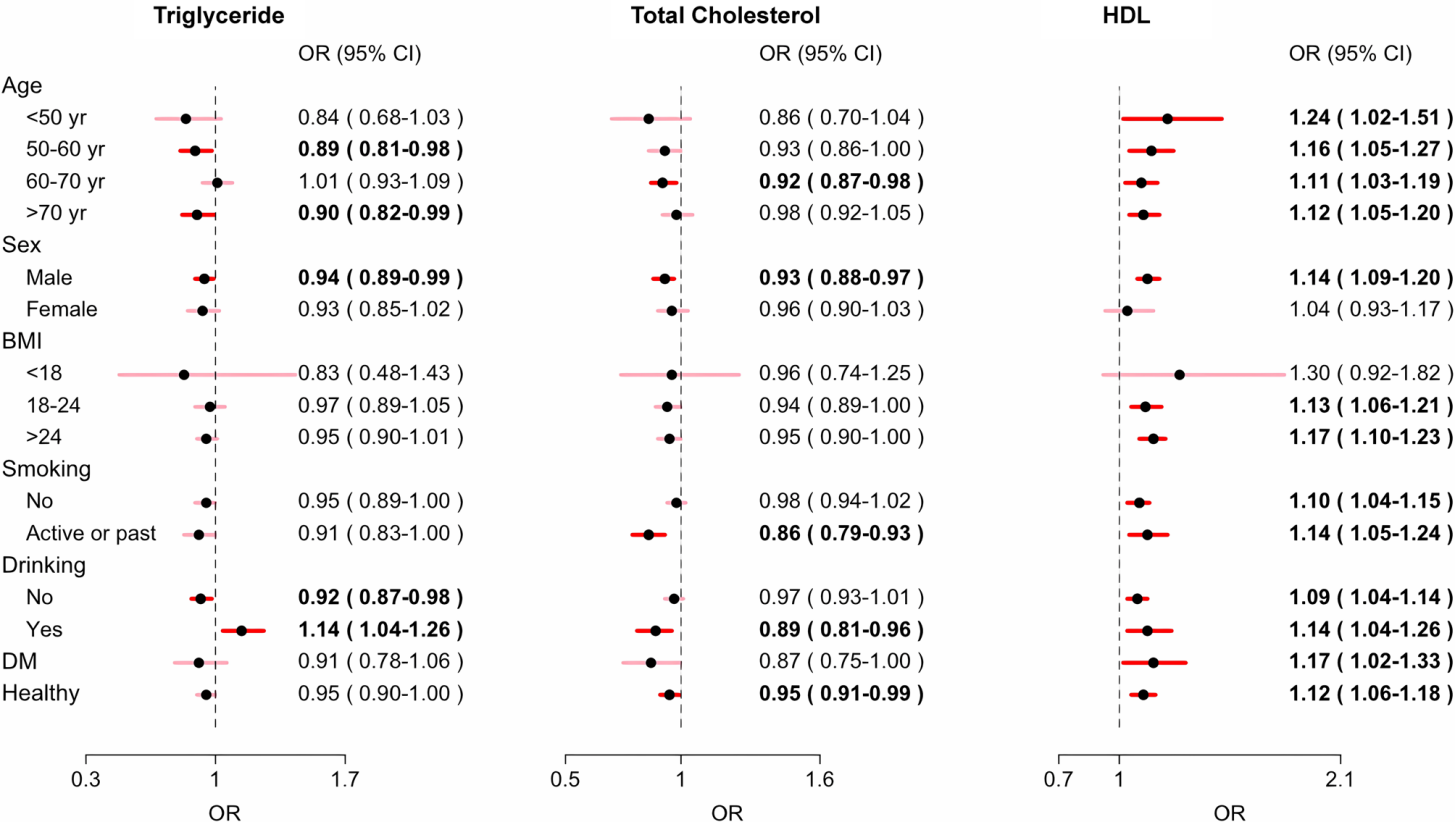
Subgroup analysis of lipid profiles and lung cancer risk from the Taiwan National Data Systems, 2012–2018 (the detailed data are presented in Supplementary Table 6). *HDL* high-density lipoprotein; *LDL* low-density lipoprotein; and *OR* odds ratio

The subgroup analysis of the associations between different lipid levels and the incidence of lung cancer is presented in Fig. 2. In terms of lung cancer risk, lower HDL cholesterol levels were associated with a greater risk of lung cancer across most subgroups, including age (30–50 years, 1.24, [1.02–1.51]; 50–60 years, 1.16 [1.05–1.27]; 60–70 years, 1.11 [1.03–1.19]; > 70 years, 1.12 [1.05–1.20]), male sex (1.14 [1.09–1.20]), body mass index (18–24, 1.13[1.06–1.21]; > 24, 1.17[1.10–1.23]), smoking status (never smoked, 1.10, [1.04–1.15]; active or past smoker, 1.14 [1.05–1.24]), diabetes status (1.17[1.02–1.33]), and participants without comorbidities (1.12[1.06–1.33]). Notably, this association was found to be nonsignificant among females (1.04 [0.93–1.17]) and individuals with a body mass index less than 18 (OR: 1.30 [0.92–1.82]).

The results of the subgroup analysis of the associations between different lipid levels and lung cancer subtypes are shown in the supplementary files and Supplementary Figs. 2–5.

### Dose‒Response Associations of Lipid Levels with Lung Cancer Risk

In the dose‒response analysis, the total cholesterol level (q2, OR: 0.94, 95% CI: 0.90–0.99; q3, OR: 0.92, 95% CI: 0.88–0.97; q4, OR: 0.90, 95% CI: 0.85–0.95, p for trend < 0.0001) and HDL cholesterol level (q2, OR: 0.91, 95% CI: 0.87–0.96; q3, OR: 0.88, 95% CI: 0.84–0.92; q4, OR: 0.87, 95% CI: 0.83–0.92, p for trend < 0.0001) revealed a dose‒response association with lung cancer development. However, triglyceride levels (q2, OR: 1.14, 95% CI: 1.09–1.19; q3, OR: 1.08, 95% CI: 1.03–1.14; q4, OR: 1.02, 95% CI: 0.97–1.08, p for trend 0.7623) and LDL cholesterol levels (q2, OR: 0.98, 95% CI: 0.94–1.03; q3, OR: 0.98, 95% CI: 0.92–1.03; q4, OR: 0.99, 95% CI: 0.93–1.06, p for trend 0.661) did not reveal a dose‒response association (Supplementary Fig. 6).

According to the results of the restricted cubic spline regression analyses, both HDL cholesterol levels (*P* nonlinearity < 0.0001) and total cholesterol levels (*P* nonlinearity = 0.0473) exhibited nonlinear relationships with a lower lung cancer risk (Supplementary Fig. 7a and b). Specifically, HDL cholesterol levels were nonlinearly associated with a lower risk of squamous cell carcinoma (*P* nonlinearity < 0.0001) (Supplementary Fig. 7c). Furthermore, triglyceride levels were nonlinearly associated with a greater risk of small cell lung cancer (*P* nonlinearity = 0.0016) (Supplementary Fig. 7d), whereas total cholesterol levels were associated with a lower risk of small cell lung cancer (*P* nonlinearity = 0.0011) (Supplementary Fig. 7e). Even after patients who were diagnosed with lung cancer within six months after a health examination were excluded, HDL cholesterol levels still showed a dose‒response association with both overall lung cancer risk (*P* nonlinearity = 0.0158) and squamous cell carcinoma risk (*P* nonlinearity = 0.0158) (Supplementary Fig. 8a and b).

## Discussion

Our study indicated that various lipids are linked to different levels of lung cancer risk. Specifically, a lower HDL cholesterol level is associated with an increased risk of lung cancer, thus indicating a dose‒response relationship. Additionally, hypertriglyceridemia is linked to a decreased risk of adenocarcinoma but an increased risk of small cell lung cancer. Furthermore, hypercholesterolemia is associated with a reduced risk of squamous cell carcinoma.

### HDL Cholesterol and the Risk of Lung Cancer

Lipids play a fundamental role in cellular structures, such as in the synthesis of cell membranes and in signal transduction [15]. Dysregulated lipid metabolism in cancer cells is associated with cancer progression and metastasis [16]. Compared with normal cells, cancer cells exhibit greater potential for synthesizing phospholipids and cholesterol, particularly through the uptake of exogenous fatty acids [17]. The upregulation of enzymes such as adenosine triphosphate citrate lyase and fatty acid synthase in the tricarboxylic acid cycle has been observed in patients with lung, colorectal, breast, and gastric cancers [16, 18].

HDL cholesterol is known for its antioxidative and anti-inflammatory effects [19]. In animal models, HDL cholesterol has been shown to decrease inflammation and the expression of cell adhesion markers, such as tumor necrosis factor-alpha, intercellular adhesion molecule-1, and P-selectin [20, 21]. These mechanisms may underlie the protective role of HDL cholesterol against the development of cancer. With respect to the association between HDL cholesterol and the occurrence of lung cancer, a study conducted in Germany revealed that patients with lower HDL cholesterol levels had a greater risk of cancers in the digestive and respiratory organs, skin, and hematologic systems [22]. Research has consistently shown that higher HDL cholesterol levels are associated with a reduced risk of lung cancer [23]. Our results provide evidence supporting the protective effect of HDL cholesterol, underscoring its important role in cancer prevention.

With respect to the associations between HDL cholesterol and different histologic subtypes of lung cancer, in a recent large-scale study, low HDL cholesterol levels were positively correlated with a greater risk of lung squamous cell cancer but not adenocarcinoma [24]. Another study noted statistically significant reductions in serum HDL cholesterol levels in patients with oral cancer squamous cell carcinoma [25]. In addition, this earlier study revealed that the association between metabolic syndrome and the risk of cancer was more pronounced in females and smokers. However, in our current subgroup analyses, we discovered that the impact of HDL cholesterol on cancer risk was more pronounced in male patients (Fig. 2). Our findings suggest that the male population remains an important target patient population for research on lipids.

### Total Cholesterol and the Risk of Lung Cancer

The association between total cholesterol and the risk of lung cancer was inconsistent with the findings of previous studies. In the present study, higher total cholesterol levels were related to a lower risk of lung cancer development. In a large prospective study in Korea, similar inverse associations between total cholesterol levels and liver, stomach, and male lung cancers were reported [26]. However, another study focusing on women's health revealed that high total cholesterol was associated with an increased risk of lung cancer [11]. To support our findings, one cohort study revealed a decrease in total serum cholesterol levels within two years before a cancer diagnosis [27], which might be attributed to increased cholesterol uptake by cancer cells [12]. One animal study revealed that mice with low lipoprotein levels are prone to airway inflammation, increased airway resistance, and airway collagen deposition [28]. This indirect evidence might further explain the inverse correlation between elevated total cholesterol levels and a lower risk of lung cancer development.

### Triglycerides and the Risk of Lung Cancer

In our study, we observed that hypertriglyceridemia was linked to a decreased risk of lung adenocarcinoma. However, several population studies have associated hypertriglyceridemia with an increased risk of non-small cell lung cancer [5, 11, 29, 30]. Since the association between triglycerides and the risk of lung cancer conflicts with the findings of previous studies, the possible mechanism of lung cancer development is uncertain. Obesity is a risk factor for hypertriglyceridemia [31]. An increasing body mass index was shown to have a protective effect against the incidence of lung cancer in a low-dose computed tomography screening cohort study in Taiwan and a meta-analysis [10, 32]. A meta-analysis revealed an increased risk of lung cancer among underweight patients (body mass index < 18 kg/m^2^) compared with normal or overweight patients. The dose‒response relationship also indicated that increasing body mass index significantly decreased the risk of lung cancer [10]. This indirect evidence might explain the relationship between hypertriglyceridemia and lower lung cancer risk. Another hypothesis linking lower serum triglycerides and the risk of cancer development is that in patients with an immune compromise status such as diabetes mellitus, reduced insulin secretion results in the downregulation of triglyceride synthesis. Low insulin secretion can trigger the upregulation of insulin-like growth factor-1 and the production of potential carcinogens [33]. Additionally, in one study, triglycerides were shown to be protective factors against liver cancer, and this phenomenon was linked to the expression level of diacylglycerol acyltransferase, which is associated with cell proliferation and tumor progression [34]. In another genetic study, there was a causal and negative association between serum triglycerides and the risk of cancer under the assumption of Mendelian randomization [35]. Notably, there was a relationship between small cell lung cancer and hypertriglyceridemia. The link between small cell lung cancer and hypertriglyceridemia has seldom been examined in the literature. Interestingly, hypertriglyceridemia has been reported to be an independent risk factor for the progression of small cell lung cancer [36]. The exact mechanism by which triglycerides affect the development of small cell lung cancer warrants further investigation.

### Lipids and the Risk of EGFR-Mutated Lung Cancer

The lipogenesis pathway, which is activated through cell proliferation pathways, is induced by EGFR [37]. EGFR signal activation contributes to increased cholesterol and fatty acid levels [15]. The associations between lipid levels and the incidence of EGFR-mutated lung cancer have rarely been examined in cohort studies. Our data indicated that hypertriglyceridemia and higher HDL cholesterol levels were associated with a lower incidence of EGFR-mutated lung cancer. The association between EGFR and the lipid production pathway warrants further investigation.

## Strengths and Limitations

Our study has several strengths. First, the study represents the largest cohort to date that examines the relationship between lung cancer and lipid profiles, significantly surpassing the scale of previous studies. This extensive sample size enables a more precise estimation of the association between lipids and lung cancer. Second, we meticulously controlled for several critical confounders, including anthropometric data, comorbidities, and concurrent medication use—a level of detail rarely achieved in similar previous studies. Last, we conducted sensitivity analyses, performed various statistical methods and assessed the dose‒response relationship, thereby enhancing the robustness of our findings. However, our study has certain limitations. First, it is an associative study and, as a result, cannot establish causality. Although some hypotheses may help explain the association we explored, there was no direct evidence to support it. Our aim is to provide insights that may generate hypotheses for future mechanistic or interventional studies. More compelling evidence, especially because different studies yield conflicting results, is needed to move lipid profile modification toward clinical practice and public health action. Interestingly, different lipids are associated with varying risks across distinct histological types of lung cancer, suggesting that in addition to shared carcinogenic pathways, different types of lung cancer may have unique oncogenic processes [38]. Various lipids may thus be involved in different steps, resulting in distinct lung cancer risks. Second, considering that all types of cancer can have a latent undetected period, whether lipid profile measurements precede the development of lung cancer remains ambiguous. To address this issue, we conducted a sensitivity analysis by excluding lung cancer patients diagnosed within half a year after the health examination. The results remained similar, while HDL cholesterol levels remained significant. Furthermore, the results of Cox regression analysis corroborated these findings. Third, several potential risk factors, such as occupation and family history, were absent from the databases we used. Therefore, we were unable to assess their influence on the observed associations.

## Conclusion

In conclusion, our extensive, population-based study revealed significant correlations between lipid profiles and lung cancer risk. We discovered that HDL cholesterol levels are consistently correlated with a reduced risk of lung cancer, with a dose‒response association. Additionally, triglycerides and cholesterols have different associations with squamous cell carcinoma, small cell lung cancer and adenocarcinoma. Although a statistically significant association is not indicative of causality, our findings from national cohort data can inform future directions in exploring the lipid‒lung cancer link. Our work reflects the utility of real-world health system data to generate research hypotheses on critical public health topics.

## Supplementary Information

Below is the link to the electronic supplementary material.

Supplementary file1 (DOCX 14 KB)

## Data Availability

No datasets were generated or analysed during the current study.

## Code Availability

This article and its supplementary materials contain all of the code presented in this study.
