# Supplementary material for: Lipid Levels and Lung Cancer Risk: Findings from the Taiwan National Data Systems from 2012 to 2018
Source: J Epidemiol Glob Health. 2025 Jan 30;15(1):11. doi: 10.1007/s44197-025-00351-8 (PMC11782738; doi:10.1007/s44197-025-00351-8)

For the association between lipid and adenocarcinoma, hypertriglyceridemia was associated with lower risk for those aged between 50 and 60 year old, aged above 70, male sex, overweight, non-drinker, and healthy population. Lower HDL level was associated with higher risk for adenocarcinoma among all age groups, male sex, overweight, non-drinkers, drinkers, and healthy population (**Supplementary Figure 2**).

For the association between lipid and SqCC, hypercholesterolemia was associated with lower risk for SqCC among those aged between 60-and-70-year-old, male sex, active or past smoker and never drinker. Also, lower HDL level (hypoalphalipoproteinemia) was associated with higher risk of SqCC among all age groups, male sex, overweight, non-drinkers, drinkers, and healthy population (**Supplementary Figure 3**).

For the association between lipid and small cell lung cancer, hypertriglyceridemia was associated with higher risk of SCLC among those aged above 60-year-old, male, female, normal body weight, never smoker, never drinker and healthy population. Hypercholestrolemia was associated with lower risk of SCLC among those aged between 60 and 70-year-old, male sex, normal body weight, drinkers, diabetes and healthy population (**Supplementary Figure 4**).

For the association between lipid and EGFR mutation status, hypertriglyceridemia was associated with lower risk of EGFR-mutated lung cancer among those aged 30-50, aged 50-60, male sex, normal weight (BMI 18-24), never smoker, DM and participants without comorbidities (**Supplementary Figure 5**).

**Supplementary Information**

**Supplementary Table 1** Clinical characteristics of lung cancer patients with different histological classifications recruited from the Taiwan National Data Systems, 2012-2018

|  | Lung cancer  **(N=30337)** | Adenocarcinoma  **(N=20107)** | Squamous  **(N=3958)** | Small cell  **(N=1989)** | Other cell type **(N=4283)** | P value |
| --- | --- | --- | --- | --- | --- | --- |
| **Age** | 66.6 ± 11.2 | 65.3 ± 11.1 | 70.0 ± 10.2 | 68.2 ± 10.5 | 68.6 ± 11.8 | <0.0001 |
| 30-50 yrs | 2085 (6.9) | 1607 (8.0) | 126 (3.2) | 82 (4.1) | 270 (6.3) | <0.0001 |
| 50-60 yrs | 6287 (20.7) | 4688 (23.3) | 513 (8.2) | 336 (16.9) | 750 (17.5) |  |
| 60-70 yrs | 9548 (31.5) | 6553 (32.6) | 1156 (29.2) | 695 (34.9) | 1144 (26.7) |  |
| >70 yrs | 12417 (40.9) | 7259 (36.1) | 2163 (54.7) | 876 (44.4) | 2119 (49.5) |  |
| **Male** | 16906 (55.7) | 9005 (44.8) | 3468 (87.6) | 1799 (90.5) | 2634 (61.5) | <0.0001 |
| **BMI** | 24.3 ± 4.02 | 24.3 ± 3.7 | 24.3 ± 3.9 | 24.7 ± 6.7 | 24.3 ± 3.9 | 0.0015 |
| <18 | 772 (2.5) | 467 (2.3) | 120 (3.0) | 52 (2.6) | 133 (3.1) | 0.0008 |
| 18-24 | 14203 (46.8) | 9490 (47.2) | 1836 (46.4) | 865 (43.5) | 2012 (47.0) |  |
| >24 | 15362 (50.6) | 10150 (50.5) | 2002 (50.6) | 1072 (53.9) | 2138 (49.9) |  |
| **Year of initial health examination** |  |  |  |  |  | <0.0001 |
| 2012 | 12677 (41.8) | 8092 (40.2) | 1804 (45.6) | 844 (42.4) | 1937 (45.2) |  |
| 2013 | 7059 (23.3) | 4682 (23.3) | 920 (23.2) | 447 (22.5) | 1010 (23.6) |  |
| 2014 | 4810 (15.9) | 3278 (16.3) | 568 (14.4) | 299 (15.0) | 665 (15.5) |  |
| 2015 | 2717 (9.0) | 1898 (9.4) | 295 (7.5) | 191 (9.6) | 333 (7.8) |  |
| 2016 | 1521 (5.0) | 1070 (5.3) | 184 (4.7) | 95 (4.8) | 172 (4.0) |  |
| 2017 | 1052 (3.5) | 732 (3.6) | 127 (3.2) | 78 (3.9) | 115 (2.7) |  |
| 2018 | 501 (1.7) | 355 (1.8) | 60 (1.5) | 35 (1.7) | 51 (1.2) |  |
| **Smoking** |  |  |  |  |  |  |
| No | 24311 (80.1) | 17341 (86.2) | 2528 (63.9) | 1093 (55.0) | 3349 (78.2) | <0.0001 |
| Seldom | 1115 (3.7) | 522 (3.6) | 256 (6.5) | 157 (7.9) | 180 (4.2) |  |
| < one pack per day | 3176 (10.5) | 1462 (7.3) | 749 (18.9) | 472 (23.7) | 493 (11.5) |  |
| >one pack per day | 1735 (5.7) | 782 (3.9) | 425 (10.7) | 267 (13.4) | 261 (6.1) |  |
| **Drinking** |  |  |  |  |  |  |
| No | 25085 (82.7) | 17023 (84.7) | 3030 (76.6) | 1489 (74.9) | 3543 (82.7) | <0.0001 |
| Occasionally | 4068 (13.4) | 2446 (12.2) | 694 (17.5) | 367 (18.6) | 561 (13.1) |  |
| Frequently | 1184 (3.9) | 638 (3.2) | 234 (5.9) | 119 (6.0) | 179 (4.8) |  |
| **Hypertension** |  |  |  |  |  |  |
| SBP<140 and DBP<90 mmHg | 19087 (62.9) | 12843 (63.9) | 2349 (59.4) | 1201 (60.4) | 2694 (62.9) | <0.0001 |
| SBP≥140 or DBP≥90 mmHg | 11250 (37.1) | 7264 (36.1) | 1609 (40.7) | 788 (39.6) | 1589 (37.1) |  |
| **Total cholesterol** | 194.7 ± 40.5 | 197.2 ± 40.0 | 186.9 ± 39.7 | 190.3 ± 44.0 | 192.0 ± 40.7 | <0.0001 |
| Total cholesterol<200 mg/dl | 17488 (57.7) | 11037 (54.9) | 2593 (65.5) | 1268 (63.8) | 2590 (60.5) | <0.0001 |
| Total cholesterol≥200 mg/dl | 12849 (42.4) | 9070 (45.1) | 1365 (34.5) | 721 (36.3) | 1693 (39.5) |  |
| **Triglycerides** | 136.4 ± 126.4 | 133.3 ± 104.5 | 139.1 ± 131.8 | 159.8 ± 241.2 | 137.6 ± 135.7 | <0.0001 |
| Triglycerides<200 mg/dl | 25989 (85.7) | 17385 (86.5) | 3339 (84.4) | 1573 (79.0) | 3692 (86.2) | <0.0001 |
| Triglycerides≥200 mg/dl | 4348 (14.3) | 2722(13.5) | 619 (15.6) | 416 (20.9) | 591 (13.8) |  |
| **HDL** | 52.8 ± 17.2 | 54.0 ± 17.2 | 49.4 ± 16.7 | 49.2 ± 18.8 | 51.7 ± 16.3 | <0.0001 |
| HDL≥40 mg/dl | 24422 (80.5) | 16746 (83.3) | 2874 (72.6) | 1433 (72.0) | 3369 (78.7) | <0.0001 |
| HDL<40 mg/dl | 5915 (19.5) | 3361 (16.7) | 1084 (27.4) | 556 (28.0) | 914 (21.3) |  |
| **LDL** | 113.3 ± 39.2 | 115.2 ± 39.3 | 108.2 ± 38.1 | 108.6 ± 40.6 | 111.2 ± 38.7 | <0.0001 |
| LDL<160 mg/dl | 27268 (89.9) | 17945 (89.3) | 3637 (91.9) | 1796 (90.3) | 3890 (90.8) | <0.0001 |
| LDL≥160 mg/dl | 3069 (10.1) | 2162 (10.8) | 321 (8.1) | 193 (9.7) | 393(9.18) |  |
| **Drug counts** | 2.20 ± 5.90 | 1.94 ± 5.50 | 2.49 ± 6.20 | 2.94 ± 6.58 | 2.74 ± 6.86 | <0.0001 |
| **Charlson Comorbidity Index** | 0.42 ± 0.96 | 0.39 ± 0.93 | 0.49 ± 1.05 | 0.49 ± 0.99 | 0.43 ± 0.99 | <0.0001 |
| **Diabetes mellitus** | 2289 (7.6) | 1475 (7.3) | 338 (8.5) | 169 (8.5) | 207 (4.8) | 0.016 |
| **COPD** | 2243 (7.4) | 1261 (6.3) | 418 (10.6) | 225 (11.3) | 339 (7.9) | <0.0001 |
| **Renal disease** | 496 (1.6) | 299 (1.5) | 90 (2.3) | 34 (1.7) | 73 (1.7) | 0.0047 |
| **Congestive heart failure** | 327 (1.1) | 187 (0.93) | 30 (0.8) | 57 (2.9) | 53 (1.2) | 0.0037 |
| **Myocardial infarction** | 94 (0.31) | 49 (0.24) | 22 (0.56) | 9 (0.45) | 14 (0.33) | 0.075 |
| **Peripheral vascular disease** | 145 (0.48) | 79 (0.39) | 25 (0.63) | 19 (0.96) | 22 (0.51) | 0.0021 |
| **Dementia** | 187 (0.62) | 111 (0.55) | 20 (0.51) | 16 (0.8) | 40 (0.93) | 0.0158 |

BMI, body mass index; CHF, congestive heart failure; CI, confidence interval; COPD, chronic obstructive pulmonary disease; DBP, diastolic blood pressure; HDL, high-density lipoprotein; LDL, low-density lipoprotein; and SBP, systolic blood pressure

**Supplementary Table 2** Year of initial health examination results and LDL and drug counts of the unmatched and matched participants recruited from the Taiwan National Data Systems, 2012-2018

|  | **Unmatched** | | |  | **Matched** | | |  |
| --- | --- | --- | --- | --- | --- | --- | --- | --- |
|  | **All**  **(N=4704853)** | **Lung cancer**  **(N=30337)** | **Control**  **(N=4674516)** | **p** | **All**  **(N=64128)** | **Lung Cancer**  **(N=21376)** | **Control**  **(N=42752)** | *p* |
| **Year of initial health examination** |  |  |  |  |  |  |  |  |
| 2012 | **998142 (21.2)** | **12677 (41.8)** | **985465 (21.1)** | **<0.0001** | **25575 (39.9)** | **8525 (39.9)** | **17050 (39.9)** | *1* |
| 2013 | **853100 (18.1)** | **7059 (23.3)** | **846041 (18.1)** |  | **14928 (23.3)** | **4976 (23.3)** | **9952 (23.3)** |  |
| 2014 | **774721 (16.5)** | **4810 (15.9)** | **769911 (16.5)** |  | **10626 (16.6)** | **3542 (16.6)** | **7084 (16.6)** |  |
| 2015 | **580312 (12.3)** | **2717 (9.0)** | **577595 (12.4)** |  | **6048 (9.4)** | **2016 (9.4)** | **4032 (9.4)** |  |
| 2016 | **493180 (10.5)** | **1521 (5.0)** | **491659 (10.5)** |  | **3384 (5.3)** | **1128(5.3)** | **2256 (5.3)** |  |
| 2017 | **478705 (10.2)** | **1052 (3.5)** | **477653 (10.2)** |  | **2409 (3.8)** | **803 (3.8)** | **1606 (3.8)** |  |
| 2018 | **526693 (11.2)** | **501 (1.7)** | **526192 (11.3)** |  | **1158 (1.8)** | **386 (1.8)** | **772 (1.8)** |  |
| **Diabetes mellitus** | 323862 (6.9) | 2289 (7.6) | 321573 (6.9) | <0.0001 | 4915 (7.7) | 1655 (7.7) | 3260 (7.6) | 0.5997 |
| **COPD** | 208543 (4.4) | 2243 (7.4) | 206300 (4.4) | <0.0001 | 3967 (6.2) | 1680 (7.9) | 2257 (5.3) | <0.0001 |
| **Renal disease** | 60898 (1.3) | 496 (1.6) | 60402 (1.3) | <0.0001 | 1128 (1.8) | 356 (1.7) | 772 (1.8) | 0.2025 |
| **Liver disease** | 145189 (3.1) | 793 (2.6) | 144396 (3.1) | <0.0001 | 1718 (2.7) | 594 (2.8) | 1124 (2.6) | 0.5483 |
| **Cerebrovascular disease** | 115334 (2.5) | 969 (3.2) | 114365 (2.5) | <0.0001 | 2119 (3.3) | 708 (3.3) | 1411 (3.3) | 0.8937 |
| **Congestive heart failure** | 35505 (0.75) | 327 (1.1) | 35178 (0.75) | <0.0001 | 598 (0.93) | 231 (1.1) | 367 (0.86) | 0.0156 |
| **Myocardial infarction** | 2298 (0.05) | 317 (1.04) | 1981 (0.04) | 0.204 | 224 (0.35) | 80 (0.37) | 144 (0.34) | 0.4489 |
| **Peripheral vascular disease** | 20754 (0.44) | 145 (0.48) | 20609 (0.44) | 0.3313 | 302 (0.47) | 101 (0.47) | 201 (0.47) | 0.9032 |
| **Drug counts** | **1.1 ± 4.3** | **2.2 ± 5.9** | **1.1 ± 4.3** | **<0.0001** | **2.2 ± 6.6** | **2.2 ± 5.9** | **2.3 ± 6.9** | *0.1273* |

COPD, chronic obstructive pulmonary disease

**Supplementary Table 3** Year of initial health examination, LDL and drug counts of matched lung cancer patients and controls across different histological classifications recruited from the Taiwan National Data Systems, 2012-2018

|  | **All**  **(N=39846)** | **Adenocarcinoma**  **(N=13282)** | **Control**  **(N=26564)** | ***p**** | **All**  **(N=10167)** | **Squamous**  **(N=3389)** | **Control**  **(N=6778)** | ***p**** | **All**  **(N=13986)** | **EGFR mutation**  **(N=4662)** | **Control**  **(N=9324)** | ***p**** | **All**  **(N=5139)** | **Small cell**  **(N=1713)** | **Control**  **(N=3426)** | *p** |
| --- | --- | --- | --- | --- | --- | --- | --- | --- | --- | --- | --- | --- | --- | --- | --- | --- |
| **Year of initial examination** |  |  |  | ***1*** |  |  |  | ***1*** |  |  |  | ***1*** |  |  |  | *1* |
| 2012 | **15018 (37.7)** | **5006 (37.7)** | **10012 (37.7)** |  | **4566 (44.9)** | **1522 (44.9)** | **3044 (44.9)** |  | **5364 (38.4)** | **1788 (38.4)** | **3576 (38.4)** |  | **2151 (41.9)** | **717 (41.9)** | **1434 (41.9)** |  |
| 2013 | **9285 (23.3)** | **3095 (23.3)** | **6190 (23.3)** |  | **2340 (23.0)** | **780 (23.0)** | **1560 (23.0)** |  | **3240 (23.2)** | **1080 (23.2)** | **2160 (23.2)** |  | **1170 (22.8)** | **390 (22.8)** | **780 (22.8)** |  |
| 2014 | **6840 (17.2)** | **2280 (17.2)** | **4560 (17.2)** |  | **1476 (14.5)** | **492 (14.5)** | **984 (14.5)** |  | **2400 (17.2)** | **800 (17.2)** | **1600 (17.2)** |  | **789 (15.4)** | **263 (15.4)** | **526 (15.4)** |  |
| 2015 | **4017 (10.1)** | **1339 (10.1)** | **2678 (10.1)** |  | **798 (7.9)** | **266 (7.9)** | **532 (7.9)** |  | **1431 (10.2)** | **477 (10.2)** | **954 (10.2)** |  | **474 (9.2)** | **158 (9.2)** | **316 (9.2)** |  |
| 2016 | **2301 (5.8)** | **767 (5.8)** | **1534 (5.8)** |  | **483 (4.8)** | **161 (4.8)** | **322 (4.8)** |  | **744 (5.3)** | **248 (5.3)** | **496 (5.3)** |  | **243 (4.7)** | **81 (4.7)** | **162 (4.7)** |  |
| 2017 | **1581 (4.0)** | **527 (4.0)** | **1054 (4.0)** |  | **348 (3.4)** | **116 (3.4)** | **232 (3.4)** |  | **516 (3.7)** | **172 (3.7)** | **344 (3.7)** |  | **219 (4.3)** | **73 (4.3)** | **146 (4.3)** |  |
| 2018 | **804 (2.0)** | **268 (2.0)** | **536 (2.0)** |  | **156 (1.5)** | **52 (1.5)** | **104 (1.5)** |  | **291 (2.1)** | **97 (2.1)** | **194 (2.1)** |  | **93 (1.8)** | **31 (1.8)** | **62 (1.8)** |  |
| **Diabetes mellitus** | 3005 (7.5) | 997 (7.5) | 2008 (7.6) | 0.851 | 809 (8.0) | 289 (8.5) | 520 (7.7) | 0.139 | 1012 (7.2) | 327 (7.0) | 685 (7.4) | 0.4743 | 429 (8.4) | 147 (8.6) | 282 (8.2) | 0.6687 |
| **COPD** | 2333 (5.9) | 883 (6.7) | 1350 (5.1) | <0.0001 | 770 (7.6) | 358 (10.6) | 412 (6.1) | <0.0001 | 697 (5.0) | 238 (5.1) | 459 (4.9) | 0.6404 | 377 (7.3) | 185 (10.8) | 192 (5.6) | <0.0001 |
| **Renal disease** | 624 (1.6) | 203 (1.5) | 421 (1.6) | 0.6687 | 224 (2.2) | 79 (2.3) | 145 (2.1) | 0.5346 | 203 (1.5) | 50 (1.1) | 153 (1.6) | 0.0081 | 119 (2.3) | 28 (1.6) | 91 (2.7) | 0.0217 |
| **Liver disease** | 1137 (2.9) | 389 (2.9) | 748 (2.8) | 0.5233 | 240 (2.4) | 87 (2.6) | 153 (2.3) | 0.332 | 400 (2.9) | 121 (2.6) | 279 (3.0) | 0.1844 | 132 (2.6) | 39 (2.3) | 93 (2.7) | 0.3496 |
| **Cerebrovascular disease** | 1121 (2.8) | 377 (2.8) | 744 (2.8) | 0.8304 | 459 (4.5) | 143 (4.2) | 316 (4.7) | 0.3109 | 382 (2.7) | 122 (2.6) | 260 (2.8) | 0.5573 | 211 (4.1) | 71 (4.1) | 140 (4.1) | 0.9208 |
| **Congestive heart failure** | 291 (0.7) | 107 (0.8) | 184 (0.7) | 0.212 | 141 (1.4) | 50 (1.5) | 91 (1.3) | 0.5893 | 93 (0.7) | 27 (0.6) | 66 (0.7) | 0.3773 | 66 (1.3) | 27 (1.6) | 39 (1.1) | 0.1888 |
| **Myocardial infarction** | 115 (0.3) | 37 (0.3) | 78 (0.3) | 0.7917 | 49 (0.5) | 21 (0.6) | 28 (0.4) | 0.1563 | 29 (0.2) | 10 (0.2) | 19 (0.2) | 0.8584 | 22 (0.4) | 9 (0.5) | 13 (0.4) | 0.45 |
| **Peripheral vascular disease** | 162 (0.4) | 42 (0.3) | 120 (0.5) | 0.0451 | 56 (0.6) | 23 (0.7) | 33 (0.5) | 0.218 | 56 (0.4) | 10 (0.2) | 46 (0.5) | 0.0138 | 38 (0.7) | 17 (1.0) | 21 (0.6) | 0.1345 |
| **Dementia** | 230 (0.6) | 47 (0.4) | 183 (0.7) | <0.0001 | 102 (1) | 13 (0.4) | 89 (1.3) | <0.0001 | 74 (0.5) | 16 (0.3) | 58 (0.6) | 0.0321 | 47 (0.9) | 15 (0.9) | 32 (0.9) | 0.8358 |
| **Drug counts** | **2.0 ± 5.9** | **1.9 ± 5.5** | **2.0 ± 6.1** | ***0.018*** | **2.8 ± 6.9** | **2.9 ± 6.6** | **2.7 ± 7.1** | ***0.0839*** | **1.9 ± 5.8** | **1.9 ± 5.6** | **1.9 ± 5.9** | ***0.9599*** | **2.8 ± 9.0** | **2.8 ± 6.8** | **2.7 ± 9.9** | *0.9703* |

COPD, chronic obstructive pulmonary disease

**Supplementary Table 4** Association between lipid profile and lung cancer occurrence according to sensitivity analysis after patients with lung cancer diagnosed within half a year after an adult health examination recruited from the Taiwan National Data Systems, 2012-2018, were excluded

|  | **Lung cancer (All)** | | | **Adenocarcinoma** | | | **Squamous carcinoma** | | | **Small cell** | | | **EGFR mutation** | | |
| --- | --- | --- | --- | --- | --- | --- | --- | --- | --- | --- | --- | --- | --- | --- | --- |
|  | **OR** | **95% CI** | **p value** | **OR** | **95% CI** | **p value** | **OR** | **95% CI** | **p value** | **OR** | **95% CI** | **p value** | **OR** | **95% CI** | **p value** |
| **Triglyceride**  **(mg/dl)** | 0.97 | 0.93-1.11 | 0.2368 | 0.90 | 0.84-0.97 | 0.0049 | 1.04 | 0.91-1.20 | 0.5599 | 1.40 | 1.16-1.69 | 0.0005 | 0.93 | 0.85-1.02 | 0.1375 |
| **Total Cholesterol**  **(mg/dl)** | 0.97 | 0.92-1.02 | 0.2685 | 1.00 | 0.95-1.06 | 0.9278 | 0.88 | 0.79-0.99 | 0.0284 | 0.88 | 0.74-1.03 | 0.1179 | 1.01 | 0.94-1.08 | 0.8671 |
| **HDL**  **(mg/dl)** | 1.06 | 1.01-1.10 | 0.0401 | 1.03 | 0.97-1.10 | 0.3326 | 1.12 | 1.00-1.26 | 0.0491 | 1.03 | 0.87-1.22 | 0.  7191 | 1.04 | 0.96-1.13 | 0.3292 |
| **LDL (mg/dl)** | 1.00 | 0.94-1.07 | 0.9687 | 0.98 | 0.90-1.06 | 0.5913 | 1.09 | 0.90-1.32 | 0.3747 | 1.31 | 1.01-1.71 | 0.0421 | 1.03 | 0.93-1.14 | 0.6042 |

CI, confidence interval; EGFR, epidermal growth factor receptor; HDL, high-density lipoprotein; LDL, low-density lipoprotein; and OR, odds ratio;

**Supplementary Table 5** Association between lipid profile and lung cancer risk according to Cox regression analysis from the Taiwan National Data Systems, 2012-2018

|  | **Lung cancer (All)** | | | **Adenocarcinoma** | | | **Squamous carcinoma** | | | **Small cell** | | | **EGFR mutation** | | |
| --- | --- | --- | --- | --- | --- | --- | --- | --- | --- | --- | --- | --- | --- | --- | --- |
|  | **HR** | **95% CI** | **p value** | **HR** | **95% CI** | **p value** | **HR** | **95% CI** | **p value** | **HR** | **95% CI** | **p value** | **HR** | **95% CI** | **p value** |
| **Triglyceride**  **(mg/dl)** | 0.88 | 0.85-0.91 | <0.0001 | 0.86 | 0.82-0.90 | <0.0001 | 0.92 | 0.83-1.01 | 0.0769 | 1.25 | 1.11-1.41 | 0.0003 | 0.87 | 0.83-0.93 | <0.0001 |
| **Total Cholesterol**  **(mg/dl)** | 0.98 | 0.94-1.12 | 0.2423 | 0.99 | 0.96-1.02 | 0.4411 | 0.88 | 0.82-0.95 | 0.0014 | 0.83 | 0.74-0.92 | 0.0007 | 0.98 | 0.94-1.02 | 0.2423 |
| **HDL**  **(mg/dl)** | 1.11 | 1.08-1.15 | <0.0001 | 1.08 | 1.04-1.13 | 0.0002 | 1.21 | 1.12-1.31 | <0.001 | 1.07 | 0.96-1.19 | 0.2324 | 1.13 | 1.08-1.20 | <0.0001 |
| **LDL (mg/dl)** | 1.04 | 1.00-1.09 | 0.0610 | 1.04 | 0.99-1.10 | 0.1174 | 1.01 | 0.88-1.15 | 0.9148 | 1.28 | 1.08-1.52 | 0.0048 | 1.08 | 1.02-1.16 | 0.0138 |

CI, confidence interval; EGFR, epidermal growth factor receptor; HDL, high-density lipoprotein; LDL, low-density lipoprotein; and OR, odds ratio;

**Supplementary Table 6** Subgroup analysis of lipid profiles and lung cancer risk from the Taiwan National Data Systems, 2012-2018: detailed data

|  | **Triglyceride** | | **Total Cholesterol** | | **HDL** | | **LDL** | |
| --- | --- | --- | --- | --- | --- | --- | --- | --- |
|  | **OR** | **95% CI** | **OR** | **95% CI** | **OR** | **95% CI** | **OR** | **95% CI** |
| **Age** |  |  |  |  |  |  |  |  |
| **<50 yr** | 0.84 | 0.68-1.03 | 0.86 | 0.70-1.04 | 1.24 | 1.02-1.51 | 0.94 | 0.68-1.29 |
| **50-60 yr** | 0.89 | 0.81-0.98 | 0.93 | 0.86-1.00 | 1.16 | 1.05-1.27 | 1.12 | 1.00-1.25 |
| **60-70 yr** | 1.01 | 0.93-1.09 | 0.92 | 0.87-0.98 | 1.11 | 1.03-1.19 | 0.96 | 0.88-1.05 |
| **>70 yr** | 0.90 | 0.82-0.99 | 0.98 | 0.92-1.05 | 1.12 | 1.05-1.20 | 1.05 | 0.92-1.18 |
| **Sex** |  |  |  |  |  |  |  |  |
| **Male** | 0.94 | 0.89-0.99 | 0.93 | 0.88-0.97 | 1.14 | 1.09-1.20 | 1.05 | 0.97-1.13 |
| **Female** | 0.93 | 0.85-1.02 | 0.96 | 0.90-1.03 | 1.04 | 0.93-1.17 | 0.99 | 0.90-1.09 |
| **Body mass index** |  |  |  |  |  |  |  |  |
| **<18** | 0.83 | 0.48-1.43 | 0.96 | 0.74-1.05 | 1.30 | 0.92-1.82 | 0.84 | 0.46-1.51 |
| **18-24** | 0.97 | 0.89-1.05 | 0.94 | 0.89-1.00 | 1.13 | 1.06-1.21 | 1.07 | 0.98-1.17 |
| **>24** | 0.95 | 0.90-1.01 | 0.95 | 0.90-1.00 | 1.17 | 1.10-1.23 | 1.01 | 0.94-1.10 |
| **Smoking** |  |  |  |  |  |  |  |  |
| **No** | 0.95 | 0.89-1.00 | 0.98 | 0.94-1.02 | 1.10 | 1.04-1.15 | 1.05 | 0.98-1.12 |
| **Active or past** | 0.91 | 0.83-1.00 | 0.86 | 0.79-0.93 | 1.14 | 1.05-1.24 | 0.92 | 0.80-1.06 |
| **Drinking** |  |  |  |  |  |  |  |  |
| **No** | 0.92 | 0.87-0.98 | 0.97 | 0.93-1.01 | 1.09 | 1.04-1.14 | 1.06 | 0.99-1.13 |
| **Yes** | 1.14 | 1.04-1.26 | 0.89 | 0.81-0.96 | 1.14 | 1.02-1.33 | 0.87 | 0.75-1.01 |
| **Diabetes mellitus** | 0.91 | 0.78-1.06 | 0.87 | 0.75-1.00 | 1.17 | 1.02-1.33 | 0.79 | 0.96-1.04 |
| **Healthy** | 0.95 | 0.90-1.00 | 0.95 | 0.91-0.99 | 1.12 | 1.06-1.18 | 1.03 | 0.97-1.10 |

**Supplementary legends**

**Supplementary Fig. 1** Linkage of different databases in Taiwan, including the National Adult Health Examination Database, the Taiwan Cancer Registry and the National Health Insurance database

**Supplementary Fig. 2** Subgroup analysis of lipid profiles and adenocarcinoma risk from the Taiwan National Data Systems, 2012-2018. HDL = high-density lipoprotein; LDL = low-density lipoprotein; and OR = odds ratio

**Supplementary Fig. 3** Subgroup analysis of lipid profiles and squamous cell carcinoma risk from the Taiwan National Data Systems, 2012-2018. HDL = high-density lipoprotein; LDL = low-density lipoprotein; and OR = odds ratio

**Supplementary Fig. 4** Subgroup analysis of lipid profiles and small cell lung cancer risk from the Taiwan National Data Systems, 2012-2018. HDL = high-density lipoprotein; LDL = low-density lipoprotein; and OR = odds ratio

**Supplementary Fig. 5** Subgroup analysis of lipid profiles and EGFR-mutated lung cancer risk from the Taiwan National Data Systems, 2012-2018. HDL = high-density lipoprotein; LDL = low-density lipoprotein; OR = odds ratio; and EGFR = epidermal growth factor receptor

**Supplementary Fig. 6** Dose‒response associations between lipid profiles and lung cancer risk from the Taiwan National Data Systems, 2012-2018. HDL = high-density lipoprotein; LDL = low-density lipoprotein

**Supplementary Fig. 7** Restricted cubic spline regression to investigate the potential associations between key lipid parameters and the incidence of lung cancer from the Taiwan National Data Systems, 2012-2018. 7a and 7b exhibited nonlinear relationships with lower lung cancer risk by increasing HDL (*P* nonlinearity<0.0001) and total cholesterol levels (*P* nonlinearity=0.0473); 7C presented a nonlinear association with a lower risk of squamous cell carcinoma by increasing HDL cholesterol levels (*P* nonlinearity<0.0001); 7D revealed that triglyceride levels were nonlinearly related to a greater risk of small cell lung cancer (*P* nonlinearity=0.0016); and 7E revealed that total cholesterol levels were associated with a lower risk of small cell lung cancer (*P* nonlinearity=0.0011). HDL = high-density lipoprotein

**Supplementary Fig. 8** Restricted cubic spline regression to investigate the potential association among key lipid parameters, and lung cancer incidence after diagnoses of lung cancer within six months following a health examination from the Taiwan National Data Systems, 2012-2018, were excluded. 8A and 8B disclosed that HDL cholesterol levels indicated a dose‒response association with both overall lung cancer risk (*P* nonlinearity=0.0158) and squamous cell carcinoma risk (*P* nonlinearity=0.0158). HDL = high density lipoprotein


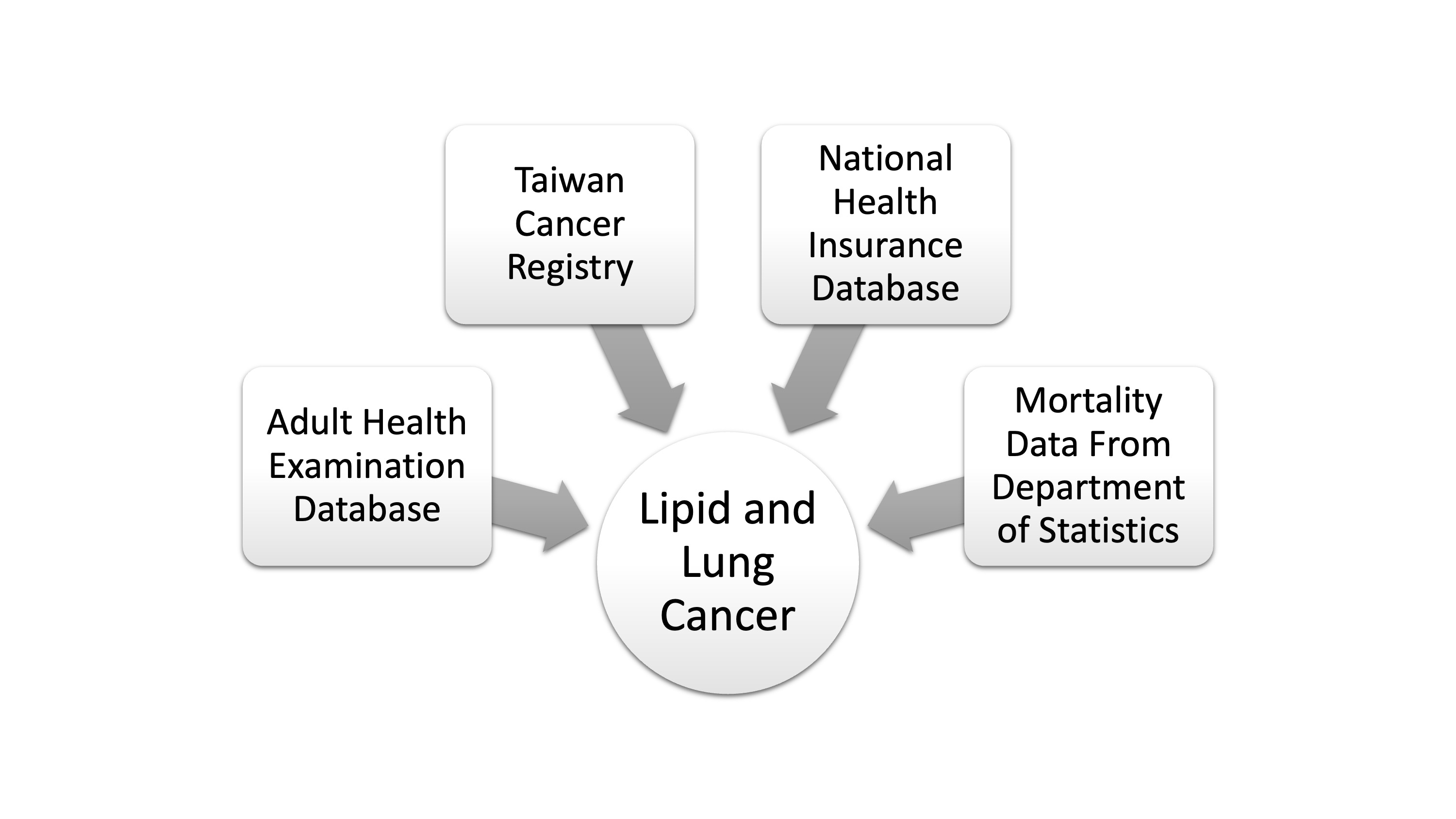

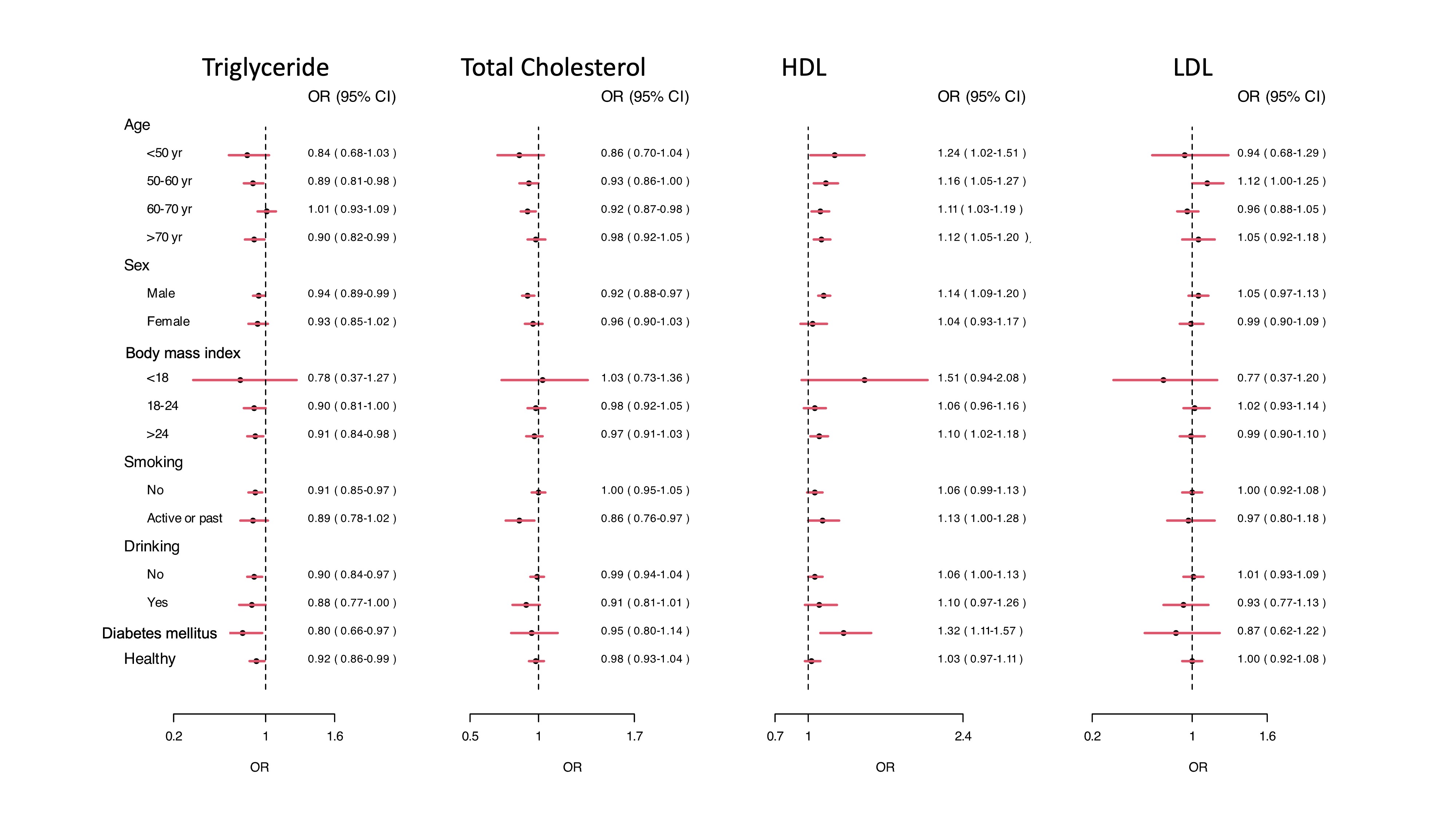

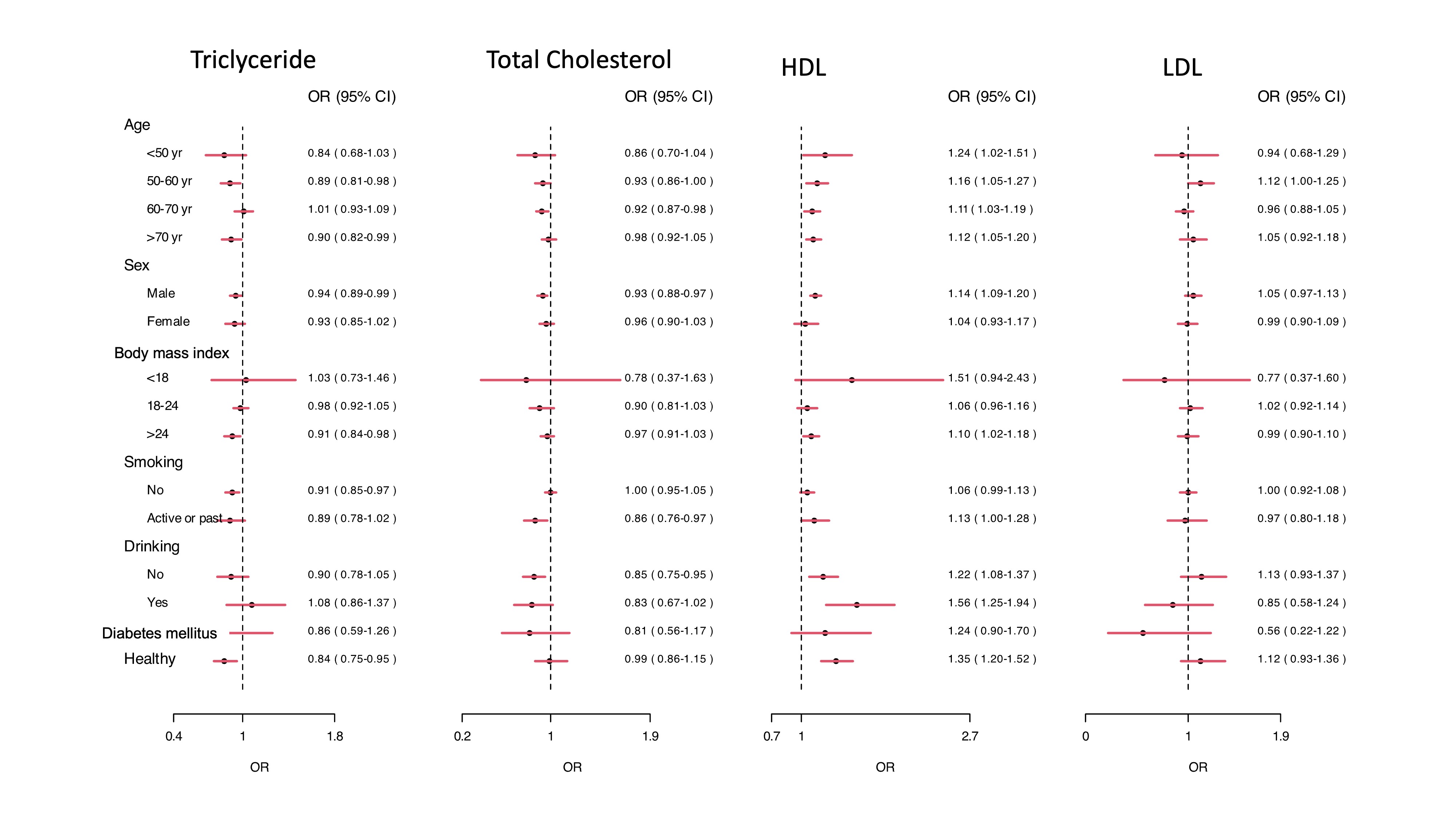

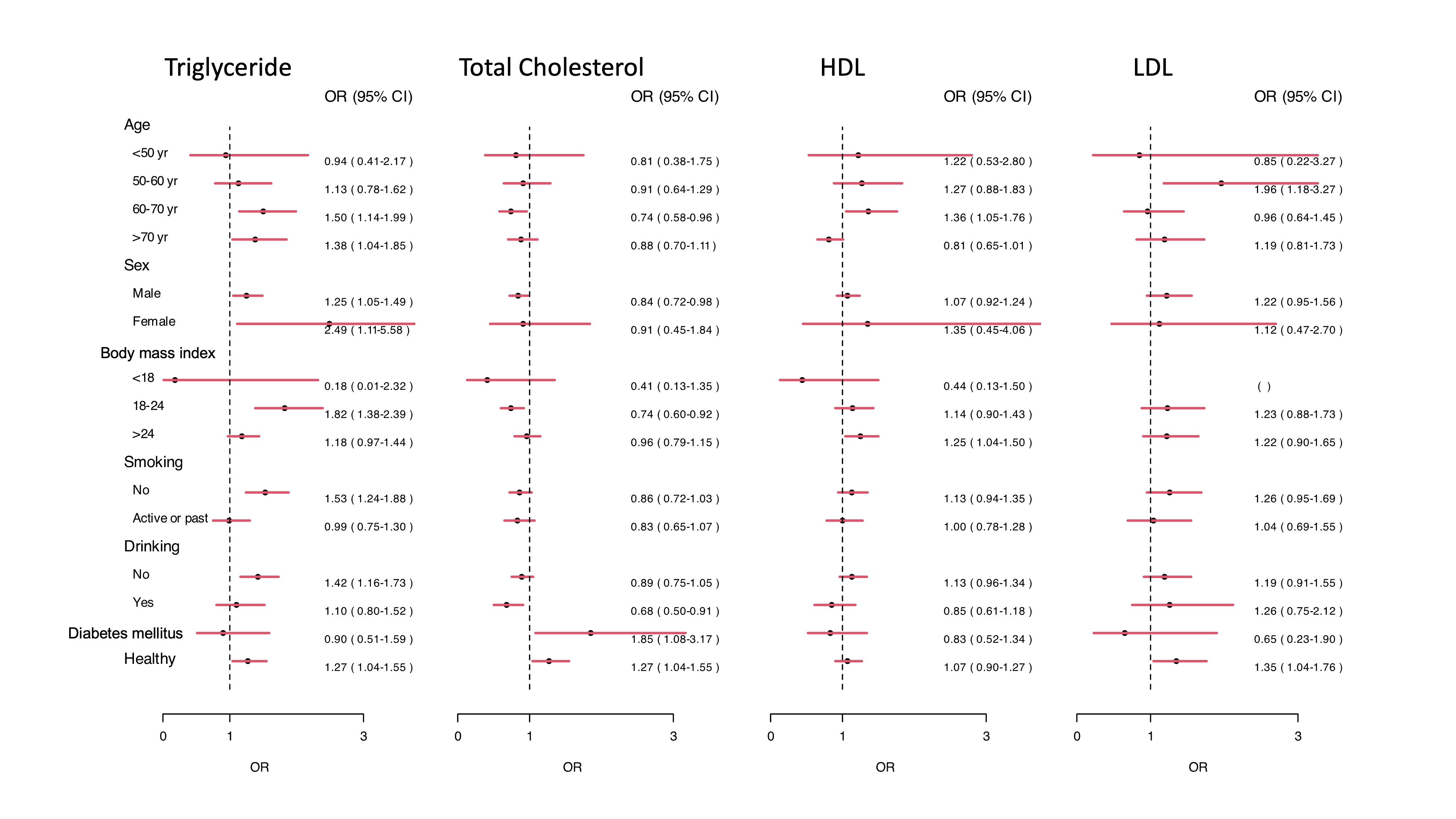

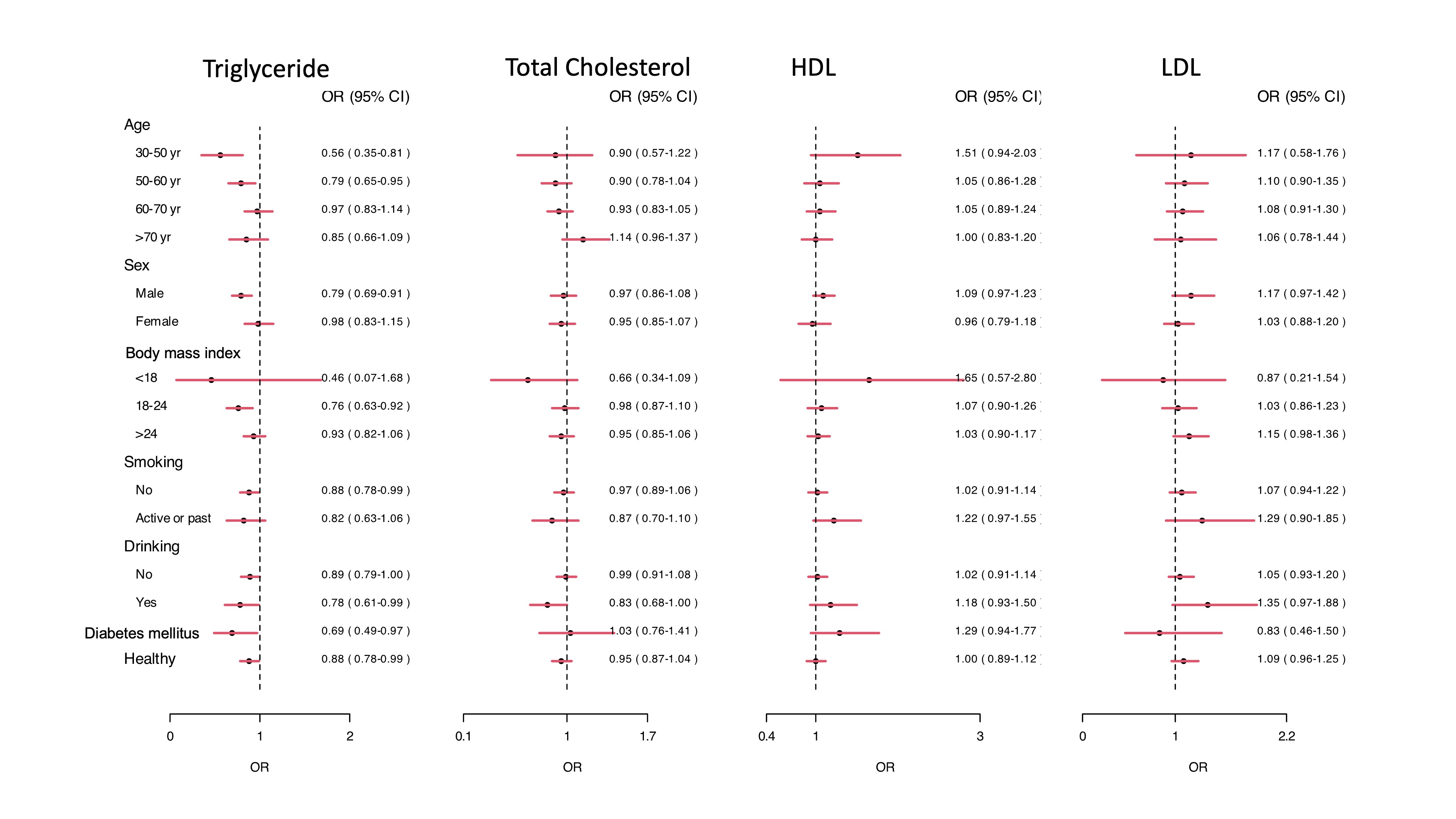

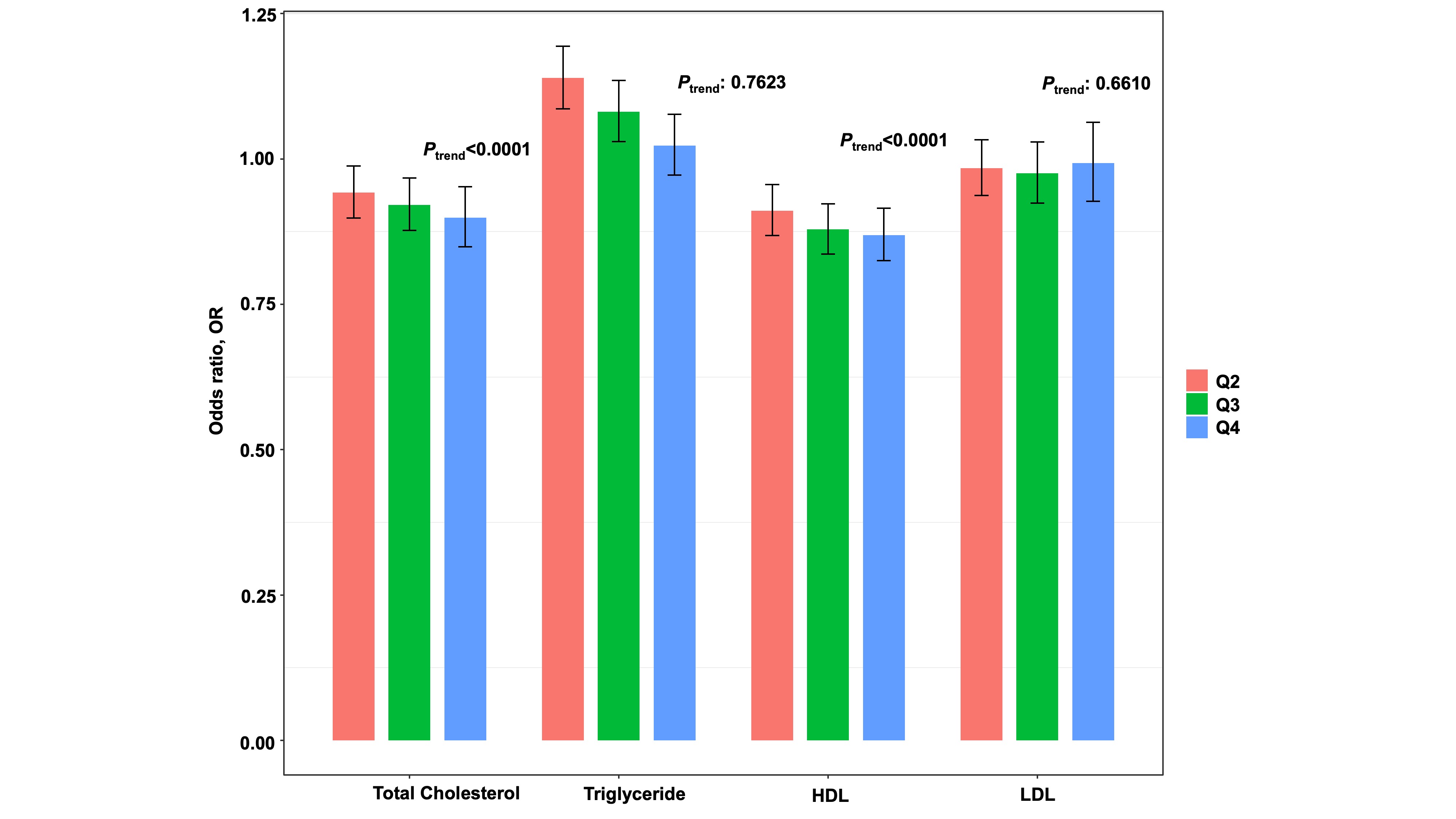

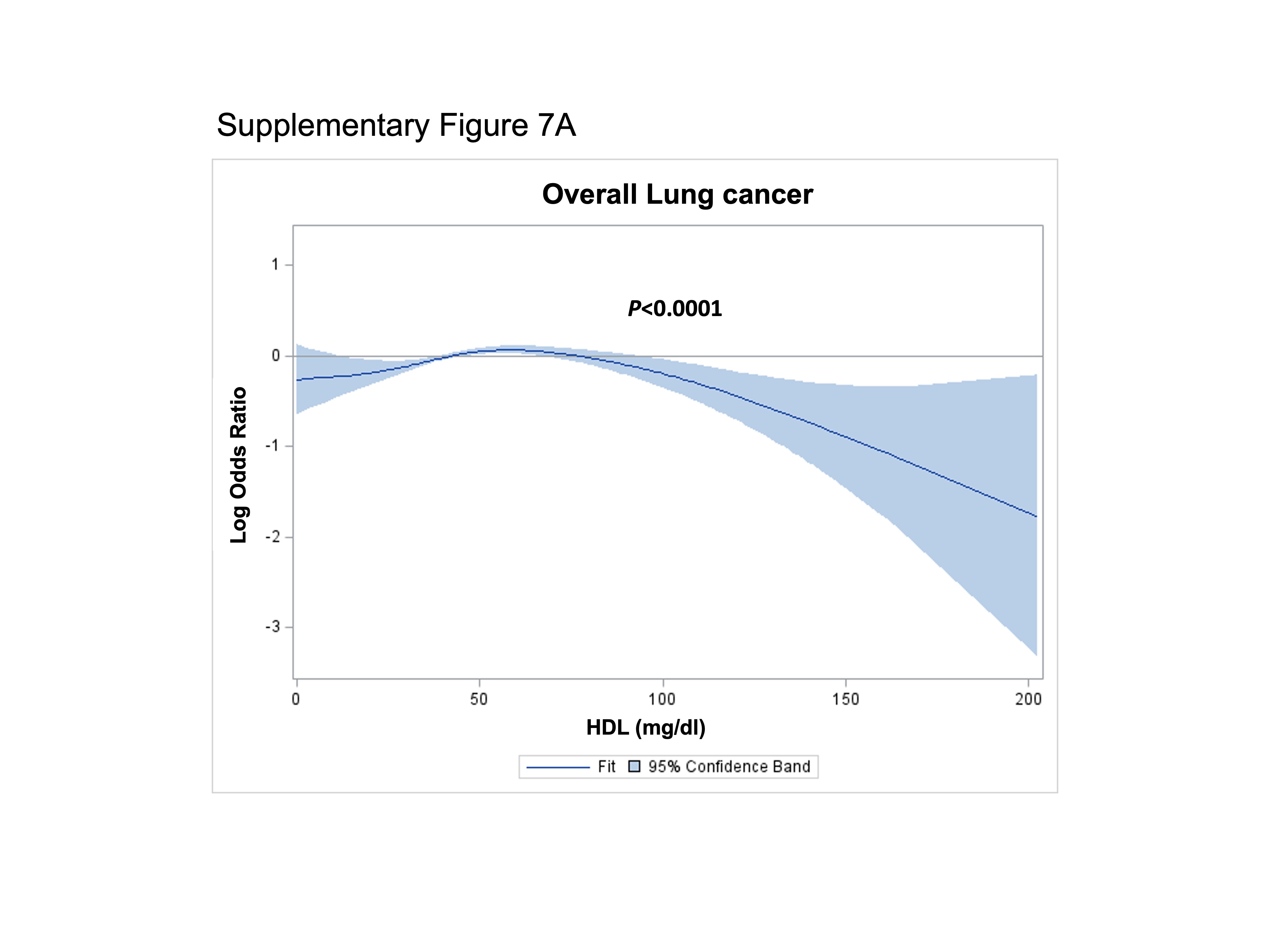

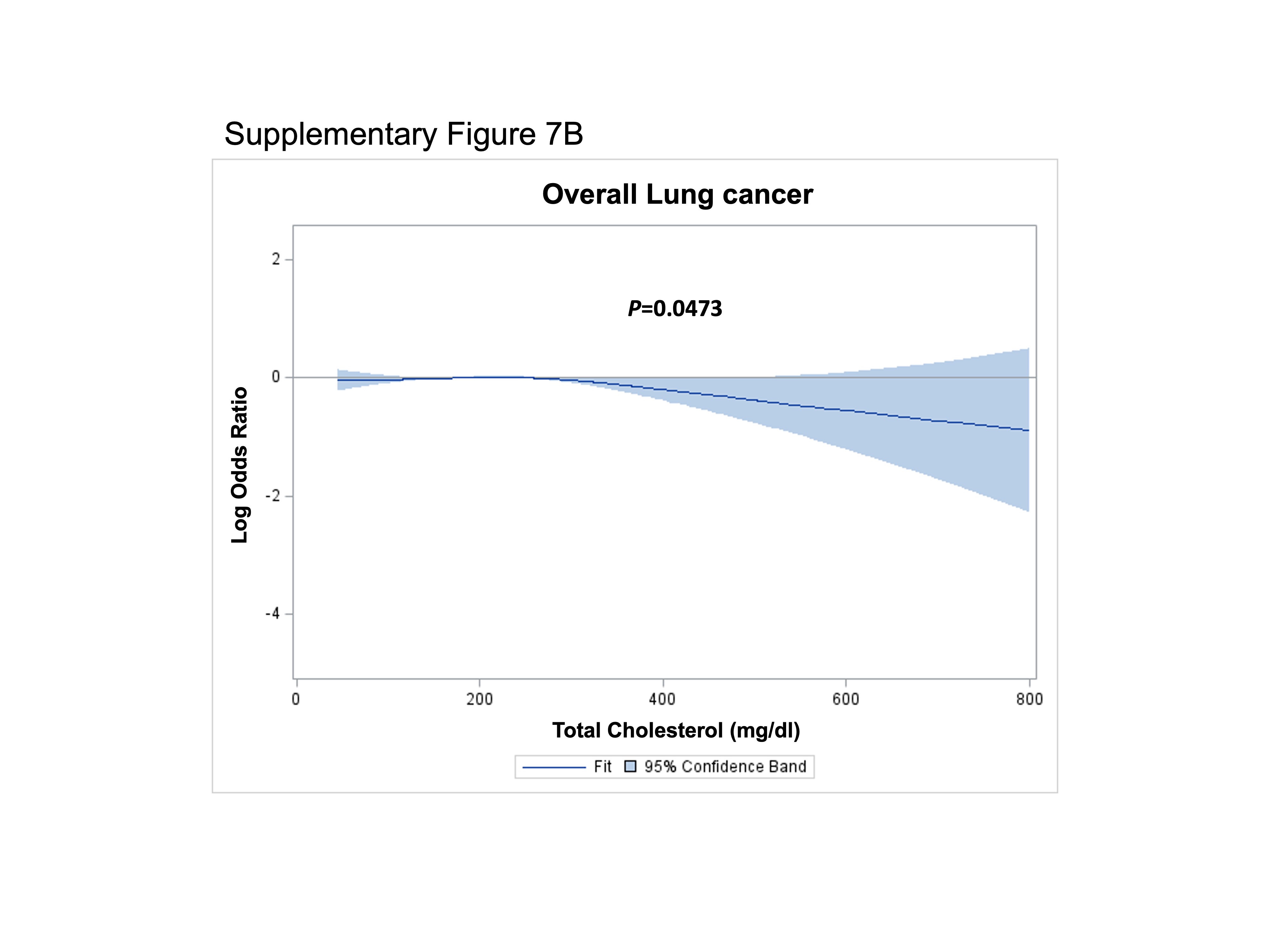

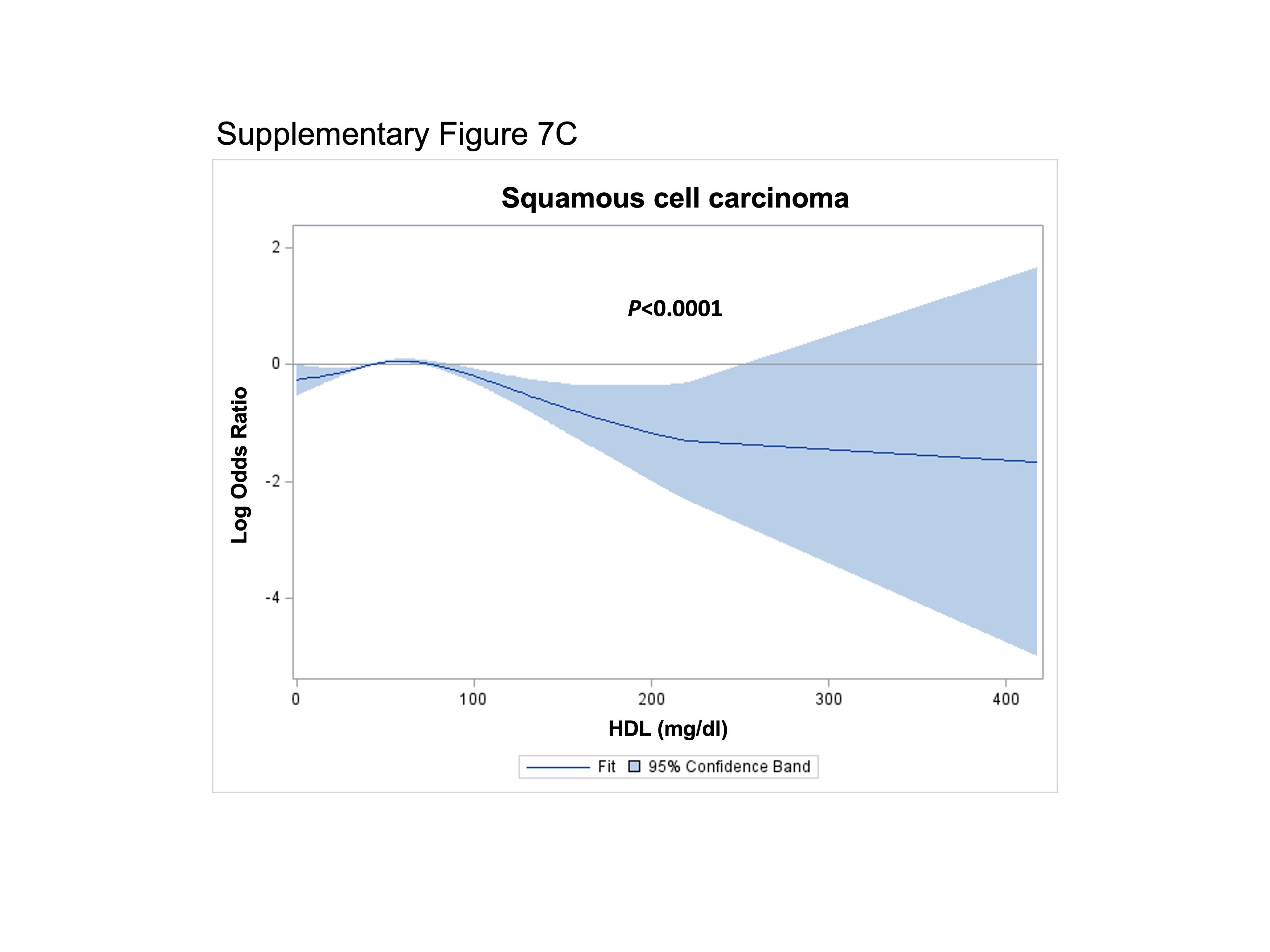

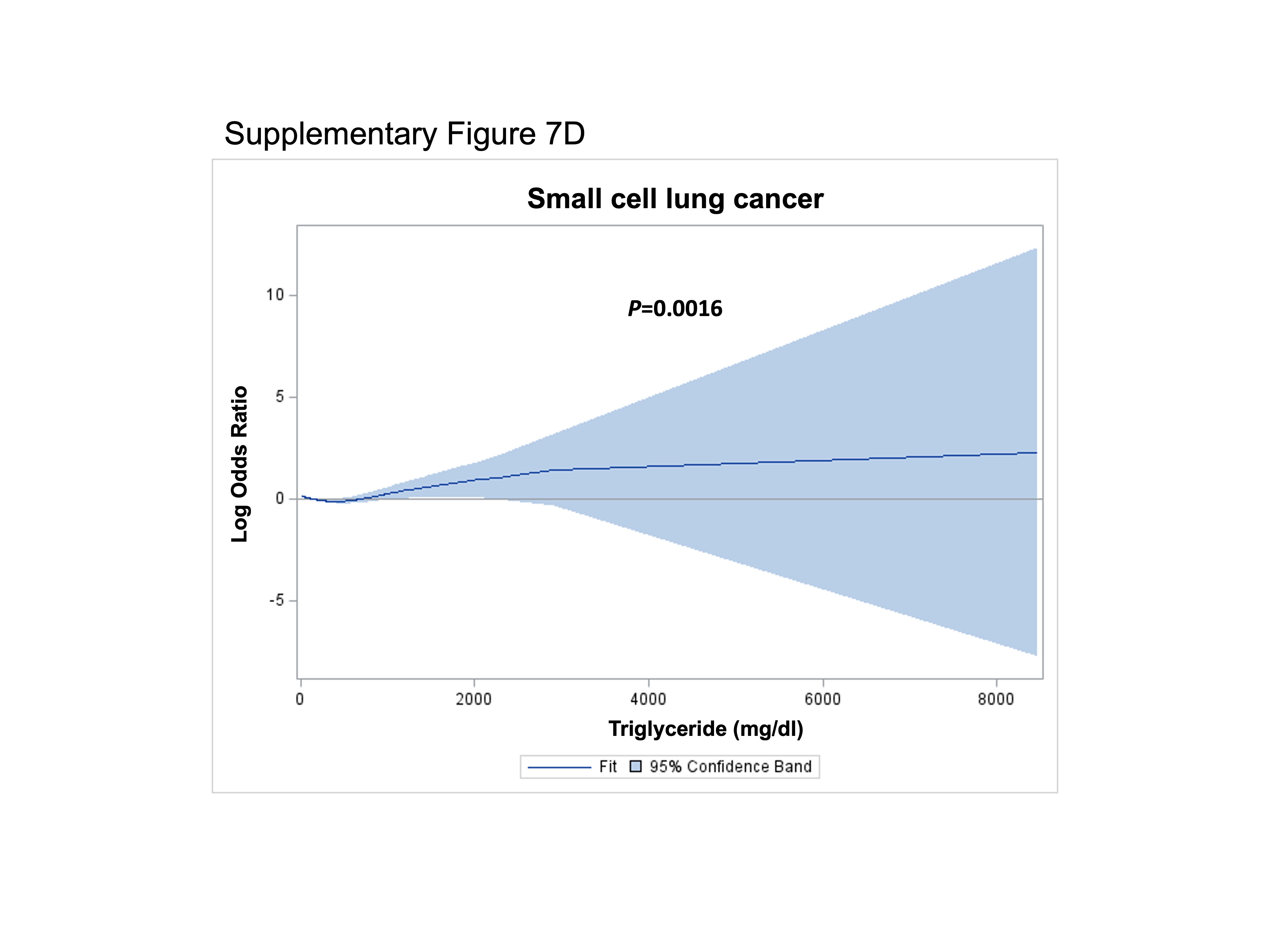

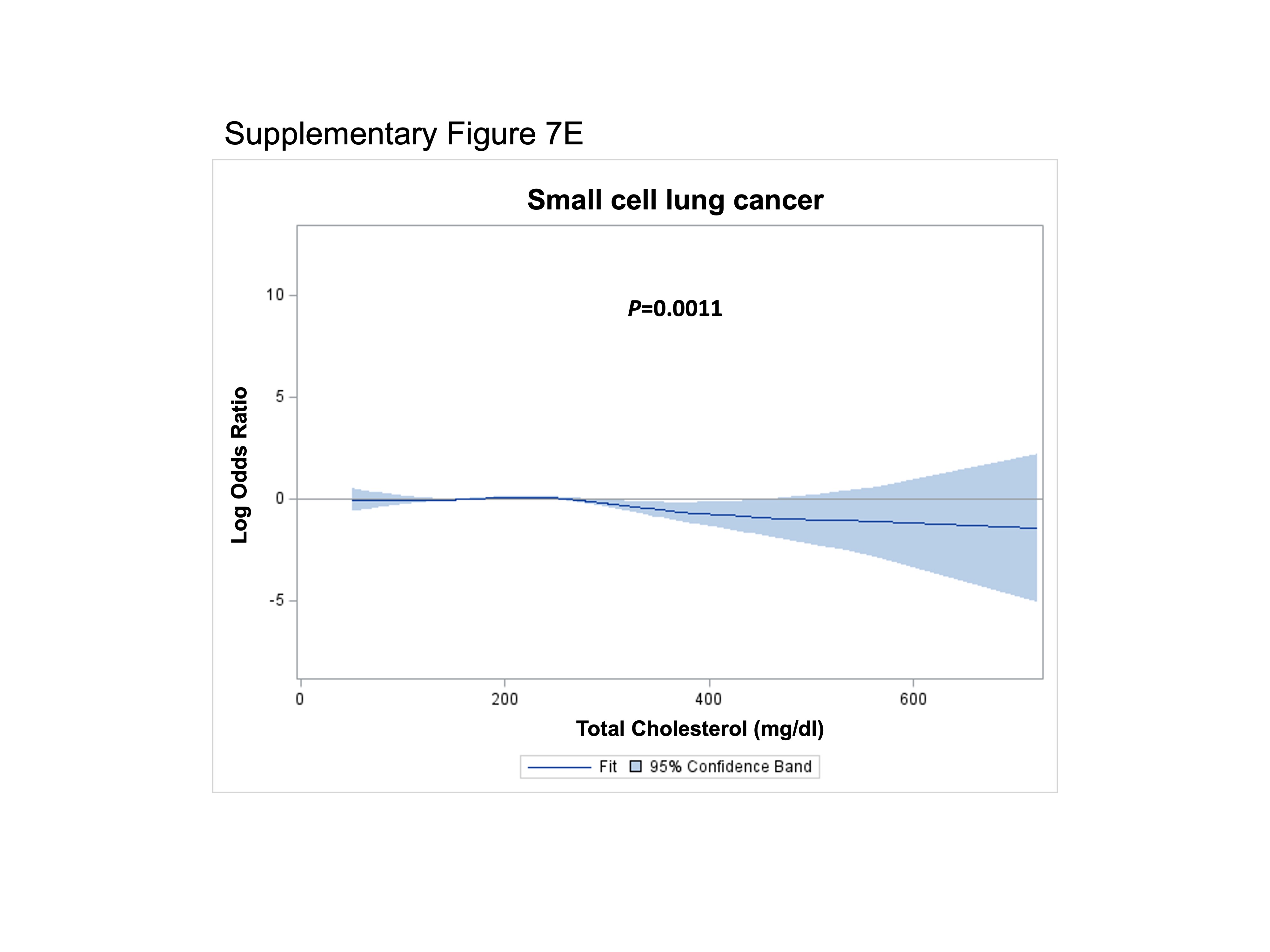

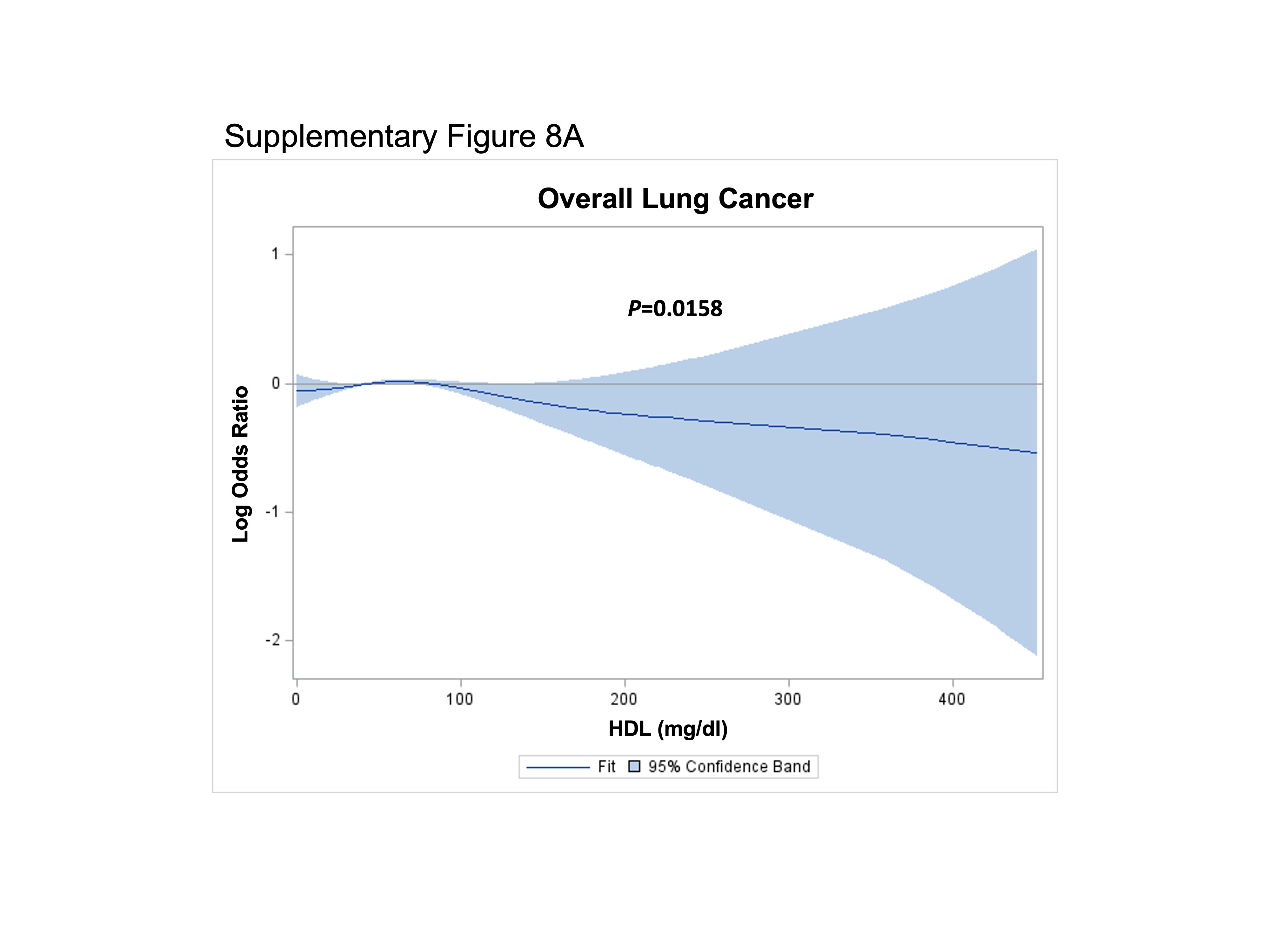

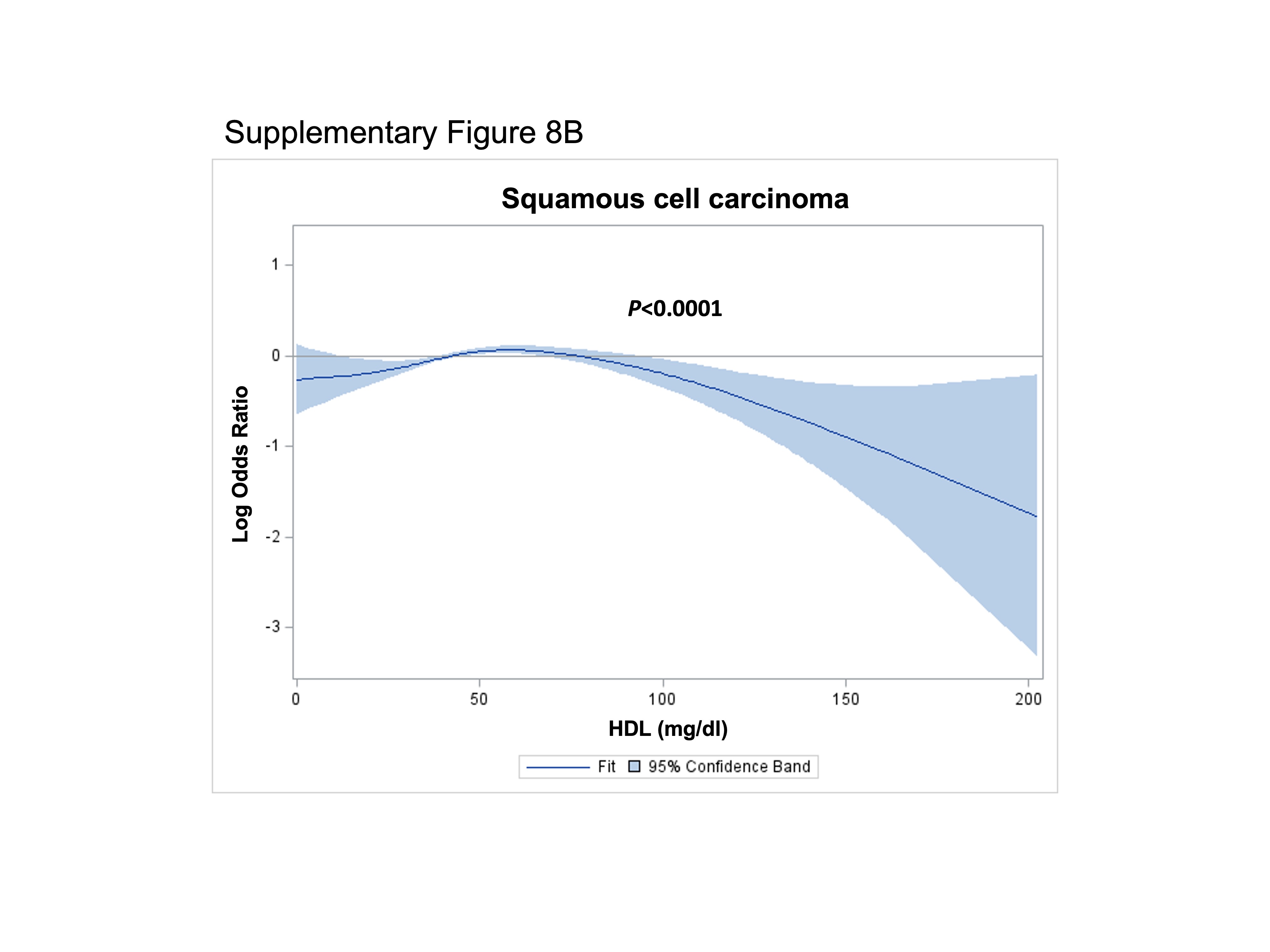

Supplement: Supplementary file 1 — Supplementary file1 (DOCX 14 KB) [file 44197_2025_351_MOESM1_ESM.docx]
